# The Synthesis, Antimicrobial Activity, and Molecular Docking of New 1, 2, 4-Triazole, 1, 2, 4-Triazepine, Quinoline, and Pyrimidine Scaffolds Condensed to Naturally Occurring Furochromones

**DOI:** 10.3390/ph15101232

**Published:** 2022-10-07

**Authors:** Ameen Ali Abu-Hashem, Sami A. Al-Hussain

**Affiliations:** 1 Heterocyclic Unit, Photochemistry Department, National Research Centre, Dokki, Giza 12622, Egypt; 2 Chemistry Department, Faculty of Science, Jazan University, Jazan 45142, Saudi Arabia; 3 Department of Chemistry, Faculty of Science, Imam Mohammad Ibn Saud Islamic University (IMSIU), Riyadh 13318, Saudi Arabia

**Keywords:** furochromones, visnagenone, khellinone, 1, 2, 4-triazole, 1, 2, 4-triazepine, quinoline, furochromenopyrimidines, triazolopyrimidines, molecular docking, antimicrobial activity

## Abstract

This study aims to synthesize a new series of furochromone derivatives, evaluate their antimicrobial properties, and improve the permeability of potent compounds to inhibit different types of bacteria and fungi. Hence, Substituted furo[3,2-*g*]chromene-6-carbonitrile (**3a,b**) readily form 7-amino-5-methyl-furo [3,2-*g*]chromene-6-carbonitrile (**4a**,**b**) via reduction using sodium borohydride in methanol. The same compounds of (**4a**,**b**) were used as starting materials for the synthesis of new furochromone derivatives such as furochromeno [2,3-*d*]pyrimidines, *N*- (6-cyano- 5-methyl-furochromene) acetamide, *N*-(6-cyano-5-methyl-furo chromene)-2-phenyl acetamide, *N*- (6-cyano-5-methyl-furochromene) formimidate, furochromeno[1,2,4]triazepin-5-amine, furochrom ene-6-carboxamide, furochromeno[1,2,4]triazolopyrimidines, and furochromeno[2,3-*b*]quinolin- 6-amine. The structures of the new compounds were determined using spectroscopy: Nuclear Magnetic Resonance (^1^H, ^13^C), Mass spectra, Infrared, and elemental analysis. Molecular docking studies were conducted to investigate the binding patterns of the prepared compounds against DNA-gyrase (PDB 1HNJ). The results displayed that compounds furochromenotriazolopyrimidine (**20a**,**b**), furochromenoquinolin-6-amine (**21a**,**b**), furochromenotriazepin-amine (**9a**,**b**), and furo- chromenopyrimidine-amine (**19a**,**b**) were excellent antimicrobials.

## 1. Introduction

**Figure 1:**
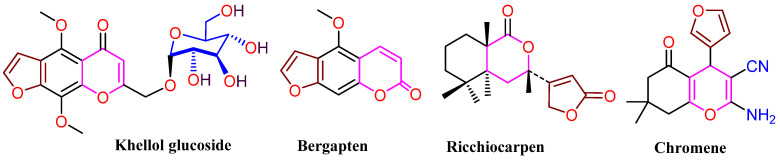
Furochromones and chromene derivatives with biological and pharmacological activities.

Natural furochromones (visnagin and khellin) and their derivatives are extracted from many plants, the most famous of which is the *Ammi visnaga* Lam plant. Previous studies have shown that furochromone compounds have broad biological activities [1,2]. Furochromone derivatives have been widely used in modern medicine to treat many ailments such as vitiligo and hair loss [2], urolithiasis and hypertriglyceridemia [3], spasms and kidney stones [4], and pain associated with renal colic [5]. They are also known to have antioxidant, antidiabetic, antispasmodic, antimutagenic, herbicidal, larvicidal, insecticidal, immunostimulatory, cardiovascular, antigastric, antineoplastic, anti-anaphylactic, anti-atherosclerotic [3], cytotoxic [6], analgesic and anti-inflammatory [7,8], antimicrobial [9,10], antiviral [11], and anticancer activity [12,13]. In addition, they have the ability to bind to DNA [14] and act as coronary vasodilators [15]. Additionally, the pyranopyrimidine moiety appears to be a significant building block in synthesized bioactive compounds with antimicrobial [16], antigenotoxic [17], anti-inflammatory, analgesic, and antiphlogistic [18,19], antiplatelet, and antithrombotic activity [20]. Benzopyran derivatives have a wide range of biological activities, including stimulating the central nervous system [21], coronary vasodilating [15,22], anti-atherosclerotic and antiatherogenic [23], antiallergic [24], spasmolytic [5], reducing blood pressure and acting as a diuretic [25], and as an antibiotic [26]. Recently, benzopyran derivatives have been used to reduce β-amyloid accumulation in Alzheimer’s disease [27]. Moreover, chromene moieties are used as a building block in the generation of natural products showing antibacterial, molluscicidal, antiallergic, antibiotic, antitumor, hypolipidemic, and immunomodulating activities [28,29,30]. In order for chromene derivatives to be of use in pharmaceuticals, great effort has been concentrated on developing new synthetic approaches like 4*H*-chromene moieties designed by polymers to support palladacycles with allenes, alkenes, and microwave-assisted liquid phase, and solid phase such as KFealumina [31]. Therefore, many heterocyclic compounds are formed, such as furochromones (visnagin and khellin) and pyran derivatives, which have various biological activities, including compounds such as Khellol glucoside, Bergapten, Ricchiocarpen, and chromene derivatives that possess molluscicidal activity [32] (Figure 1).

In our previous work, we synthesized a new class of novel heterocyclic compounds from natural furochromones and other compounds, with the study of the biological activity of these compounds [1,7,8,9,10,12,14,33,34,35]. The present work presents a new technique for the synthesis of furochromene-6-carbonitrile, furochromenopyrimidinone, furochromeno[2,3-e][1,2,4]triazepin-5-amine, furochromeno[3,2-e][1,2,4]triazolopyrimid- ine, and furochromenoquinolin-6-amine using new reagents and rapid, convenient procedures that produce better yields and a higher purity of the products than the conventional methods.

## 2. Results and Discussion

### 2.1. Synthesis

Furochromones such as visnagin (**1a**) and khellin (**1b**) were hydrolyzed with aqueous potassium hydroxide, giving visnagenone (**2a**) or khellinone (**2b**), respectively [12,14]. Moreover, treatment of (**2a**) and (**2b**) with malononitrile in ethanol and the addition of small amounts of triethylamine gives 7-imino-(4-methoxy or 4,9-dimethoxy)- 5-methyl- 7*H*-furo [3,2-*g*] chromene-6-carbonitrile (**3a**,**b**).

**Scheme 1:**
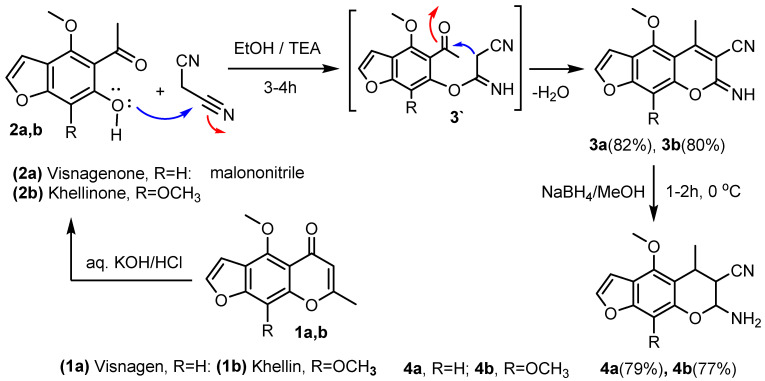
Synthesis of 7-imino/7-amino-furo[3,2-*g*]chromene-6-carbonitrile derivatives from furochromones.

Furthermore, the same compounds (**3a**,**b**) were reduced using sodium borohydride in methanol [36] to produce 7-amino-(4-methoxy or 4,9-dimethoxy)-5-methyl-6,7-di- hydro-5*H*-furo [3,2-g]chromene-6-carbonitrile (**4a,b**), respectively. The infrared spectra of (**3a**) and (**3b**) showed absorption bands at *ν* 3300–3310 cm^−1^ indicative of the (NH) groups and at *ν* 2235–2240 cm^−1^ suggestive of carbonitrile (CN) groups. The (^1^H) Nuclear Magnetic Resonance spectrum of (**3a**) displayed a singlet signal at *δ* 9.50 ppm corresponding to one proton (NH) group, which was D_2_O exchangeable. The (^1^H)-NMR spectrum of (**4b**) exhibited one singlet signal at *δ* 8.45 ppm, corresponding with a two-proton (NH_2_) group with exchangeable D_2_O. The MS of (**3a**), (**3b**), (**4a**), and (**4b**) revealed molecular ion peaks at *m*/*z* 254 (M^+^, 100%), 284 (M^+^, 98%), 258 (M^+^, 100%), and 288 (M^+^, 95%) respectively (Scheme 1).

The synthesis of (**5a**,**b**) was preceded by an initial condensation of the (NH_2_) function in (**4a**,**b**) with the (C=O) group in formamide via intermediate (**5′a**,**b**), which, cyclized by the nucleophilic addition of the (NH_2_) function into the cyano group, produced (6-methoxy or 6,10-dimethoxy)-5-methyl-furo[3′,2′:6,7]chromeno[2,3-*d*] pyrimidin-4-amine (**5a**,**b**). An infrared spectrum of (**5a**,**b**) demonstrated absorption bands at *ν* 3425–3422 cm^−1^ indicative of an amino group, with a deficiency in the distinctive absorption of the cyano group. The (^1^H)-NMR spectrum of (**5a**) showed a singlet signal at *δ* 6.45 ppm, matching the two protons of the (NH_2_) group with exchangeable D_2_O.

Moreover, the addition of (**4a**) or (**4b**) to formic acid yielded (6-methoxy or 6, 10-dimethoxy)-5-methyl-furochromeno [2,3-*d*]pyrimidin-4-one (**6a**,**b**). ^1^H-NMR spectrum of (**6a**) exhibited a singlet signal at *δ* 10.70 ppm, indicating one proton of the (NH) group with exchangeable D_2_O.

However, compounds (**4a**) and (**4b**) reacted with acetic anhydride and the product obtained depended on the reaction conditions. 

Thus, the treatment of (**4a**) or (**4b**) with acetic anhydride under reflux in the absence of pyridine gave *N*-(6-cyano-(4-methoxy or 4,9-dimethoxy)-5-methyl-5*H*-furo[3,2-*g*] chromen-7-yl)acetamide (**7a**) or (**7b**)**,** respectively.

When (**4a**) or (**4b**) was reacted with acetic anhydride in pyridine in a water bath, it produced (6-methoxy or 6, 10-dimethoxy)-2,5-dimethyl-furochromeno [2,3-*d*]pyrimidin- 4-ol, (**8a**) or (**8b**), the same compounds obtained from the reflux of (**7a**) or (**7b**) in a pyridine solution. An infrared spectrum of (**7a**) displayed an absorption band at *ν* 2225 cm^−1^ due to the cyano group and 1691 cm^−1^ for the carbonyl group. The (^1^H)-NMR spectrum of (**7a**) exhibited one singlet signal at *δ* 9.35 ppm, conforming to one proton (NH) group. Also, the IR spectrum of (**8a**) displayed the absence of the absorption band (CN) group and the presence of absorption bands at *ν* 3412 cm^−1^ for the hydroxyl group. The (^1^H)-NMR spectrum of (**8a**) exposed a singlet signal at *δ* 12.10 ppm, matching to the proton (OH) group, which was D_2_O exchangeable.

Moreover, compounds (**7a**) and (**7b**) underwent cyclization by refluxing with hydrazine hydrate (N_2_H_4_) in a mixture of ethanol with a catalytic amount of piperidine to produce (7-methoxy or 7,11-dimethoxy)-2,6-dimethyl-furochromeno[2,3-e][1,2,4]triazepin-5-amine (**9a/b**). An infrared spectrum of (**9a**,**b**) exposed an absorption band at *ν* 3420–3390 cm^−1^ indicative of the (NH_2_) group and an absorption band at *ν* 3305–3301 cm^−1^ for the (NH) group. The (^1^H)-NMR spectrum of (**9a**) exhibited singlet signals at *δ* 6.85 and 10.10 ppm, matching two-proton (NH_2_) and one-proton (NH) group with exchangeable D_2_O, respectively.

Furthermore, the partial hydrolysis of compounds (**4a**) and (**4b**) was carried out by stirring with concentrated sulfuric acid to give 7-amino-(4-methoxy or 4,9-dimethoxy) -5-methyl-5*H*-furo [3,2-*g*]chromene-6-carboxamide (**10a/b**). The ^1^H NMR spectrum of (**10a**) showed two deuterium oxide exchangeable singlets at *δ* 6.86 and 7.25 ppm conforming to (NH_2_) and (CONH_2_) protons, respectively. Furthermore, acid-catalyzed nucleophilic cyclo-condensation of amino-carboxamide (**10a/b**) with acid chlorides, namely benzoyl chloride in glacial acetic acid, produced (6-methoxy or 6, 10-dimethoxy)—5-methyl-2- phenyl-furochromeno[2,3-*d*]pyrimidin-4-one(**11a**,**b**). Also, the (^1^H)-NMR spectrum of (**11a**) showed a singlet signal at *δ* 11.10 ppm matching to the proton of (NH), and showed two exchangeable deuterium oxides. 

**Scheme 2:**
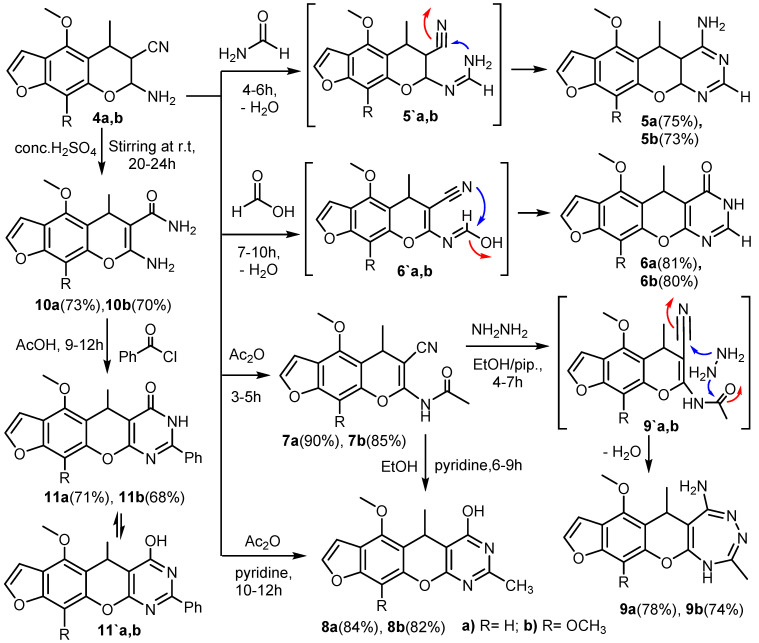
Synthesis of furochromeno[2,3-*d*]pyrimidinone and furochromeno[2,3-e][1,2,4] triazepin-5-amine derivatives.

The MS spectra of (**9a**), (**9b**), (**10a**), (**10b**), (**11a**), and (**11b**) demonstrated molecular ion peaks at *m*/*z* 312 (M^+^, 90%), 342 (M^+^, 85%), 274 (M^+^, 100%), 304 (M^+^, 92%), 360 (M^+^, 100%), and 390 (M^+^, 94%), respectively. Assignment of the newly prepared compounds was based on spectroscopic analyses such as infrared, ^1^H-NMR, ^13^C-NMR, mass spectra, and elemental analyses (cf. Scheme 2).

The reaction of the amino group in compounds (**4a**,**b**) with carbon-disulfide through a nucleophilic attack of the thiol group on the carbonitrile (CN) function formed the intermediate (**12′a**,**′b**), and rearrangement of (**12″a**,**″b**) produced the pyrimidine dithione derivatives (**12a**,**b**). Hence, refluxing of (**4a/b**) with carbon disulfide in pyridine produced (6-methoxy or 6, 10-dimethoxy)-5-methyl-furochromeno[2,3-*d*]pyrimidine-dithione (**12a/b**). The IR spectra of (**12a**) and (**12b**) exhibited the absence of absorption bands determined to be amino and cyano groups and showed absorption bands at *ν* 3235–3210 cm^−1^ for (2NH) groups, and at *ν* 1300–1295 cm^−1^ for (2C=S) functions. The (^1^H)-NMR spectrum of (**12a**) showed singlet signals at *δ* 9.10 and 12.02 ppm, corresponding with the two protons of the (2NH) groups with exchangeable D_2_O.

Additionally, heating of (**4a/b**) with 2-phenylacetyl chloride in pyridine yielded *N*-(6-cyano-(4-methoxy or 4, 9-dimethoxy)-5-methyl-furochromene)-2-phenylacetamide (**13a,b**)**,** respectively.

Moreover, the latter compounds’ (**13a**,**b**) treatment with hydrogen peroxide was followed via hydrolysis with sodium hydroxide [34] to produce 2-benzyl-(6-methoxy or 6,10-dimethoxy)-5-methyl-furochromeno[2,3-*d*]pyrimidin-4-one (**14a/b**) with an inter- mediate (**14′a/′b**). The infrared spectra of (**13a**,**b**) showed absorption bands at *ν* 3220–3215 cm^−1^ for (NH), 2244–2240 cm^−1^ for (CN), and 1692–1690 cm^−1^ for the carbonyl group. The IR spectrum of (**14a/b**) revealed the disappearance of the absorption band recognized in the (CN) group. The (^1^H)-NMR spectrum of (**14a**) revealed a singlet signal at *δ* 9.50 ppm, matching the proton of the (NH) group, which was D_2_O exchangeable.

Likewise, the refluxing of compounds (**4a**,**b**) with phenyl-isothiocyanate or phenyl- isocyanate in pyridine produced 4-imino-(6-methoxy or 6,10-dimethoxy)-5-methyl -3-phenyl-furochromeno[2,3-*d*]pyrimidine-2-thione (**15a** or **15b**) and 4-imino-(6-methoxy or 6,10-dimethoxy)-5-methyl-3-phenyl-furochromeno[2,3-*d*]pyrimidin-2-one (**16a**,**b**), respectively.

The infrared spectra for (**15a/b**) and (**16a/b**) displayed the absence of an absorption bands assignable to cyano and amino functional groups and compounds (**15a/b**) and (**16a/b**) showed absorption bands at *ν* 3280–3225 cm^−1^ for (2NH), 1335–1332 cm^−1^ for (C=S) functions, and 1688–1682 cm^−1^ for carbonyl groups, respectively. The ^1^HNMR spectra of (**15a**) exhibited singlet signals at *δ* 9.10 and 9.60, corresponding to the two protons of the (2NH) groups with exchangeable D_2_O. 

**Scheme 3:**
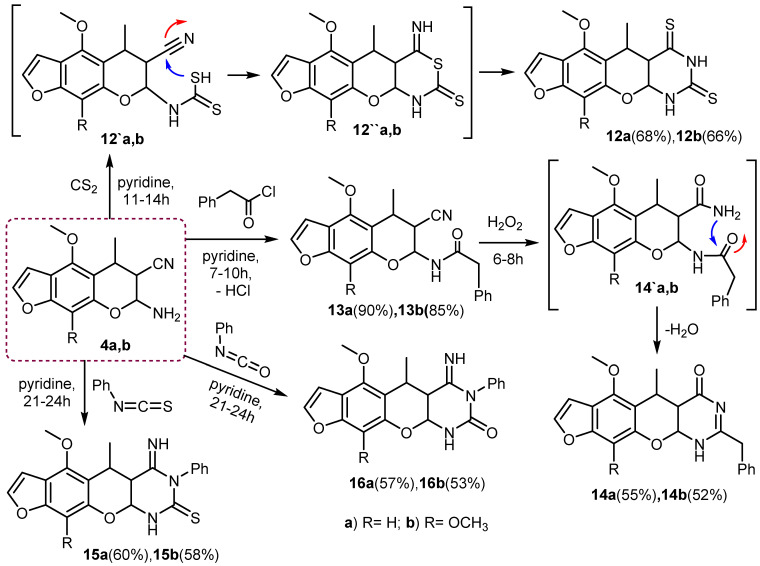
Synthesis of furochromeno[2,3-*d*]pyrimidinone/pyrimidine-thione derivatives.

The mass spectra of (**14a**), (**14b**), (**15a**), (**15b**), (**16a**), and (**16b**) presented molecular ion peaks at *m*/*z* 376 (M^+^, 77%), 406 (M^+^, 70%), 393 (M^+^, 80%), 423 (M^+^, 75%), 377 (M^+^, 72%), and 407 (M^+^, 69%), respectively. The newly prepared compounds were confirmed based on elemental analyses such as IR, ^1^H-NMR, ^13^C-NMR, and mass spectra, as shown in Scheme 3.

Likewise, the refluxing and condensation of (**4a/b**) with triethylorthoformate in acetic anhydride gives the corresponding ethyl *N*-(6-cyano-(4-methoxy or 4,9- dimethoxy) -5-methyl-furo [3,2-*g*]chromen-7-yl)formimidate (**17a**,**b**). 

The IR spectra of (**17a**,**b**) exhibited the absence of any absorption due to amino groups and the presence of a cyano function at *ν* 2220–2218 cm^−1^; the ^1^HNMR spectrum of (**17a**) showed the presence of triplet and quartet signals at *δ* 1.25 and 3.66 ppm due to the ethoxy group.

Whereas the latter compound (**17a**,**b**) produced (6-methoxy or 6, 10-dimethoxy)-3, 5-dimethyl-furochromeno[2,3-*d*]pyrimidin-4-imine(**18a,b**)-upon treatment with methyl- amine in refluxing absolute ethanol at room temperature, the (^1^H)-NMR spectrum of (**18a**) revealed a singlet broad signal at *δ* 9.65 ppm, conforming to the proton of (NH), D_2_O exchangeable.

Also, the same compounds (**17a**,**b**) underwent further cyclization upon reaction with hydrazine hydrate in absolute ethanol at room temperature under refluxing and stirring to give 4-imino-(6-methoxy or 6,10-dimethoxy)-5-methyl-furochromeno[2,3-*d*]pyrimidin- 3(5*H*)-amine (**19a**,**b**). The infrared spectrum of (**19a**,**b**) displayed the absence of a cyano function with absorption bands of the amino group at *ν* 3425–3422 cm^−1^ and of the (NH) group at *ν* 3260–3258 cm^−1^. The (^1^H)-NMR spectrum of (**19a**) showed the disappearance of the ethoxy group and an absorption of singlet signals at *δ* 6.40 ppm, matching the two-proton (NH_2_) group and at *δ* 9.70 ppm for the proton (NH) group, which was D_2_O exchangeable.

In addition, the cyclization of the same compounds (**19a**,**b**) was accomplished via the reaction of (**19a**,**b**) with triethylorthoformate in acetic anhydride and refluxing to yield (12-methoxy or 8,12-dimethoxy)-13-methyl-furochromeno [3,2-e][1,2,4]triazolo [1,5- c]pyrimidine (**20a**,**b**). The infrared spectrum of (**20a**,**b**) demonstrated a disappearance of absorption bands (NH) and amino groups. The (^1^H)-NMR spectrum of (**20b**) exposed two singlet signals at *δ* 6.05 and 7.76 ppm, indicative of two protons of (CH) triazole and (CH) pyrimidine rings.

Similarly, the Friedländer synthesis is a condensation of *o*-amino-aryl with ketones in the presence of catalysis to form quinolines. 

Therefore, condensation of compounds (**4a/b**) with cyclohexanone in dry 1,2-di- chloroethane in the presence of catalyzed AlCl_3_ [37] with stirring under an argon atmosphere at room temperature causes cyclo-condensation to produce a (4-methoxy or 4,13-dimethoxy)-5-methyl-furochromeno[2,3-*b*]quinolin-6-amine (**21a/b**) intermediate, (**21′a**,**b**).

**Scheme 4:**
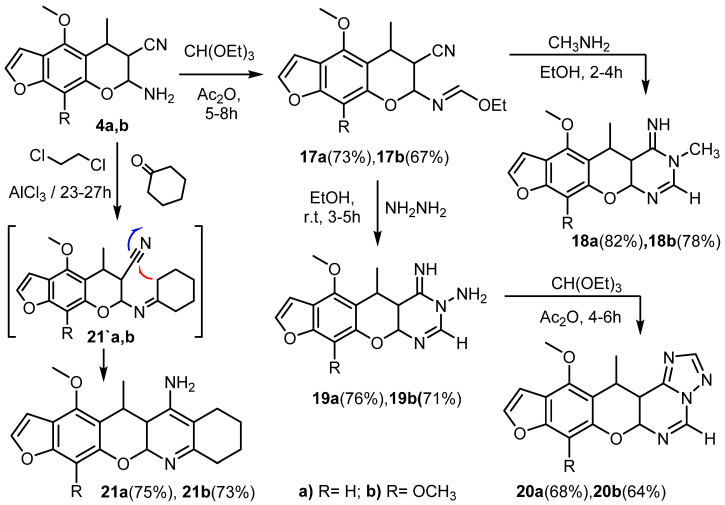
Synthesis of furochromeno[3,2-e][1,2,4]triazolo[1,5-*c*]pyrimidine and furochromeno [2,3-*b*]quinolin-6-amines.

The infrared spectra of compounds (**21a**,**b**) showed an absorption band at *ν* 3415–3412 cm^−1^, indicative of one (NH_2_) group. Moreover, the (^1^H)-NMR spectra of (**21a**) exhibited a singlet signal at *δ* 6.65 ppm, matching to the two protons of the amino group, which were D_2_O exchangeable. The MS spectra of (**19a**), (**19b**), (**20a**), (**20b**), (**21a**), and (**21b**) demonstrated molecular ion peaks at *m*/*z* 300 (M^+^, 100%), 330 (M^+^, 90%), 310 (M^+^, 85%), 340 (M^+^, 80%), 338 (M^+^, 93%), and 368 (M^+^, 90%), respectively**.** All newly prepared compounds were founded on elemental analyses, infrared, NMR (^1^H, ^13^C), and MS spectra, as shown in Scheme 4.

### 2.2. Biological Activities

#### 2.2.1. Biological Screening

All the newly synthesized compounds of the furochromone derivatives were tested in vitro for their antimicrobial activities at a minimum inhibitory concentration [9,10,33,34,35,38,39,40,41,42,43,44,45,46,47,48,49,50,51,52,53,54] versus various bacteria and fungi; the results are shown in Table 1 and Table 2. Some of these compounds showed high antimicrobial activity, comparable to that of cefotaxime sodium (MIC = 1–4 μmol mL^−1^). Compounds (**20a**,**b**), (**21a**,**b**), (**9a**,**b**), and (**19a**,**b**) exhibited potent anti- microbial activity against Gram-negative bacteria; *Klebsiella pneumoniae*, *Escherichia coli*, and Gram-positive bacteria; *Streptococcus pyogenes*, *Staphylococcus aureus.*

Further, compounds (**15a**,**b**), (**16a**,**b**)**,** (**18a**,**b**)**,** and (**12a**,**b**) revealed moderate antimicrobial activity. The MIC values in μmol mL^−1^ of these compounds were as follows: (**20a**,**b**) (1–5), (**21a**,**b**) (2–6), (**9a**,**b**) (4–7), and (**19a**,**b**) (5–9).

Compounds (**21a**,**b**), (**20a**,**b**), (**9a**,**b**), and (**19a**,**b**) also showed higher antifungal activity, with MIC in μmol/cm^3^ of (**21a**,**b**) (1–4), (**20a**,**b**) (1–5), (**9a**,**b**) (2–6), and (**19a,b**) (3–7), whose results were compared with the positive control, nystatin (MIC: 1–3 μmol mL^−1^). 

Some of the compounds revealed moderate antifungal activity when compared with nystatin (MIC 1–3 μmol mL^−1^): (**15a**,**b**) (5–8), (**16a**,**b**) (7–10), (**18a**,**b**) (9–13) and (**12a**,**b**) (11–15). The tested fungi were *Candida albicans*, *Curvularia lunata, Alternaria alternate*, and *Aspergillus niger*.

#### 2.2.2. Structural Activity Relationship (SAR)

The results show that some types of bacteria and fungi are more sensitive to the synthesized compounds; some compounds have better antimicrobial activity, such as furo[3′,2′:6,7]chromeno[3,2-e][1,2,4]triazolo[1,5-c]pyrimidine (**20a,b**); furo[3′,2′:6,7]chro- meno[2,3-b]quinolin-6-amine (**21a,b**), furo[3′,2′:6,7]chromeno[2,3-e][1,2,4]triazepin-5- amine (**9a**,**b**), and furo[3′,2′:6,7]chromeno[2,3-*d*]pyrimidin-3(5*H*)-amine (**19a**,**b**). So, there are other compounds with moderate antimicrobial activity such as furo [3′,2′:6,7] chromeno [2, 3-*d*]pyrimidine-2-thione (**15a**,**b**), furo [3′,2′:6,7]chromeno [2,3-*d*] pyrimidin- 2-one (**16a**,**b**), furo[3′,2′:6,7]chromeno[2,3-*d*]pyrimidin-4-imine (**18a**,**b**), and furo [3′,2′:6,7] chromeno [2,3-*d*] pyrimidine-dithione (**12a**,**b**).

Based on previous studies and practical results, the structure activity relationship of the compounds, with results showing good antimicrobial activity have been discussed, and the following can be confirmed:

The presence of functional groups linked with furochromones such as methyl, methoxy, amino, imino, hydroxyl, phenyl, thioxo, acetyl, 1,2,4-triazole, 1,2,4-triazepine, pyrimidine, quinoline, and fused rings; furochromenoquinoline, furochromenotriazolo- pyrimidine, furochromenotriazepine, and furochromenopyrimidine moieties; and heteroatoms such as oxygen, nitrogen, and sulfur.

Some of the functional groups present in the prepared compounds are called “activated” because they tend to donate electrons to the ring such as amine, amide, hydroxy, alkyl, alkoxy, and ester groups. Other functional groups are called “deactivated” because they tend to withdraw electrons from the ring such as cyano, carbonyl, sulfonyl, haloalkyl, nitro, and ammonium groups. Thus, the existence of these functional groups leads to lipophilic groups in diverse places on the triazepine, pyrimidine, quinoline, triazole, pyran, and phenyl rings, and hydrophobic reactions of the triazolopyrimidine, furochromenotriazepine, and furochromenoquinoline moieties at the more active site. In current work and previous studies, most types of bacteria and fungi are affected by this class of heterocyclic compounds [9,10,33,34,35,36,38,39,40,41,42,43,44,45,46,47,48,49,50,51,52,53,54].

Furthermore, previous results and our findings corroborate the promising antimicrobial activity of furochromenotriazolopyrimidine (**20a**,**b**), furochromeno- quinoline (**21a**,**b**), furochromenotriazepine (**9a**,**b**), and furochromenopyrimidine (**19a**,**b**) derivatives, which can be developed to enhance their antimicrobial activity.

### 2.3. Molecular Modeling

The structure of FabH (PDB 1HNJ) was obtained from the RCSB protein Data Bank [55]. Glide was used to analyze the interactions of the active compounds with the enzyme. All the heteroatoms were removed and isolated from the 1HNJ.pdb, to make complex receptors free of any ligand before docking. The water molecule of the enzyme was removed, and hydrogen atoms were added to the typical geometry before docking. The ligand file was submitted to the Chem3D Ultra Visualizing program to be reduced to the lowest energy and to obtain a standard 3D structure. A grid box was created with active residues of 1HNJ protein, using receptor grid generation in the glide tool of the Schrodinger suite to produce a good docking reaction at the formed binding domain. The engaged free energy of output docked complexes was studied using prime MMGBSA of the Schrodinger suite. The binding free energy demonstrated the consanguinity of H-bond and pi-sigma reactions between target 1HNJ protein and little ligand molecules. Table 3 shows eight docked complexes with an H-bond length below 3.2, suggesting that the docked complexes have steady conformation. The binding free energy of docked complexes was in the range of −38.8 to −49.84, with negative dG values designating the formation of steady complexes.

#### 2.3.1. Binding Free Energy Calculation

The XP docked output molecules are used to calculate the binding free energy of protein–ligand complexes using prime MMGBSA (molecular mechanics generalized Born surface area) at force domain OPLS-2005 [56]. Free energy of binding describes the affinity of a ligand molecule with a protein. The binding free energy was calculated at binding poses of protein–ligand complexes as follows:

∆G Binding = ∆G complex − ∆ (G protein + G ligand),

where G Binding is the Minimized binding free energy; G complex, G protein, and G ligand represent the free energy of the protein–inhibitor complex, protein, and inhibitor, respectively.

#### 2.3.2. Molecular Docking

**Figure 2:**
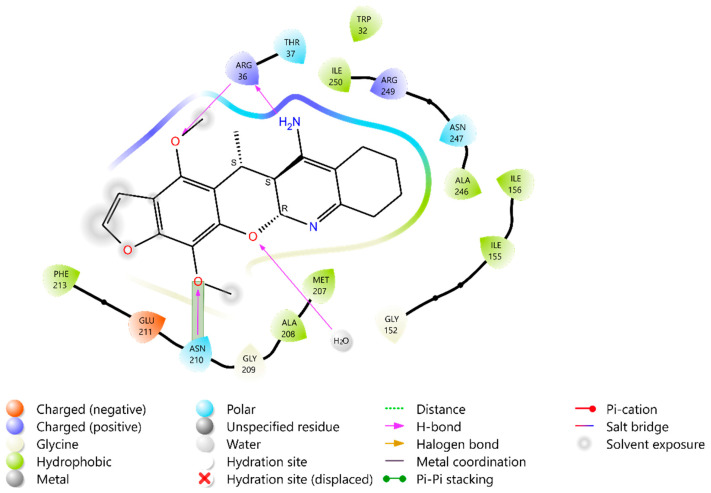

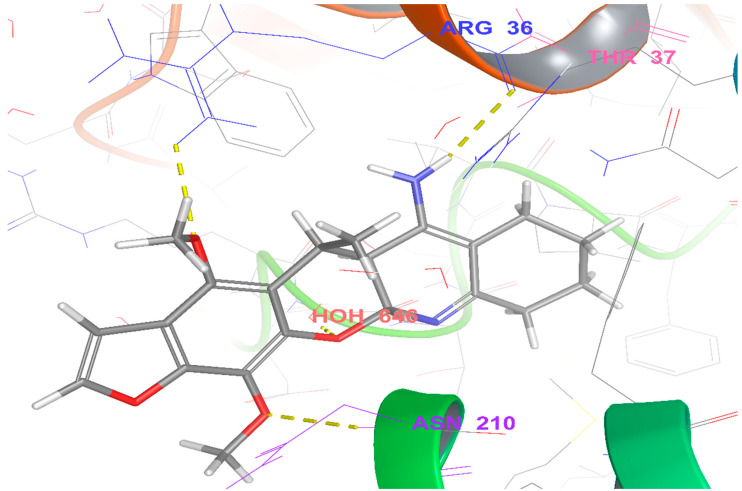
The three-dimensional docked poses of the best compound, (**21b**), with active amino acid residues of 1HNJ protein are visualized by Maestro. The two-dimensional interactions of the lead molecule are from the Schrodinger suite.

**Figure 3:**
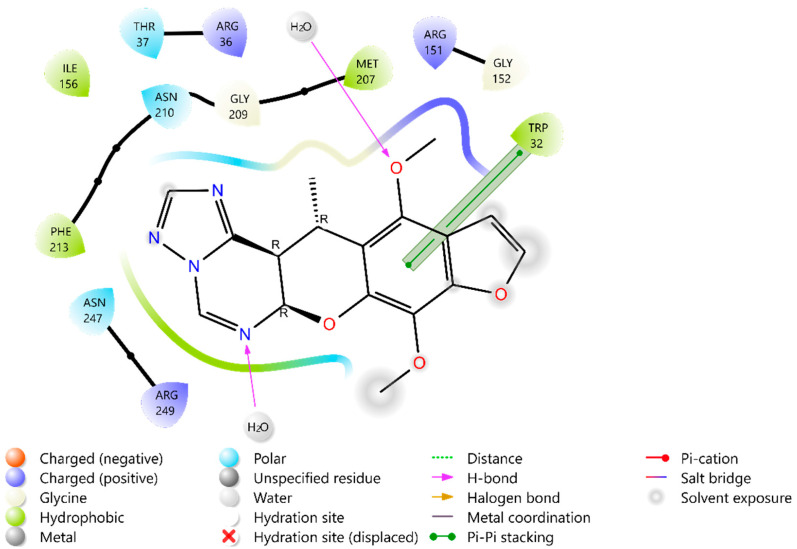

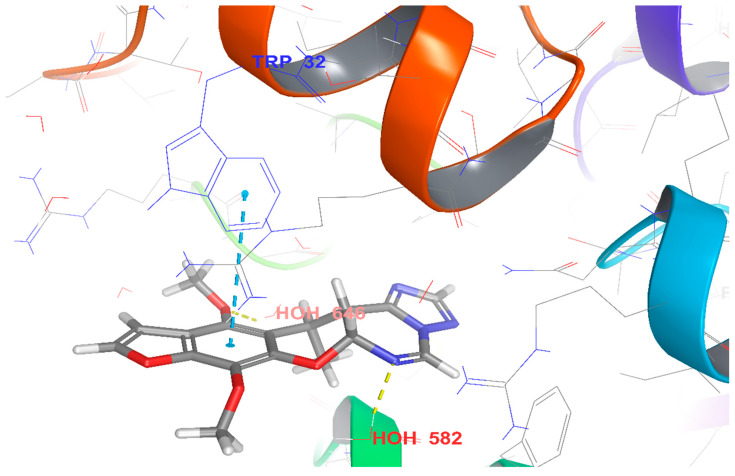
The three-dimensional docked poses of the best compound, (**20b**), with active amino acid residues of 1HNJ protein.

**Figure 4:**
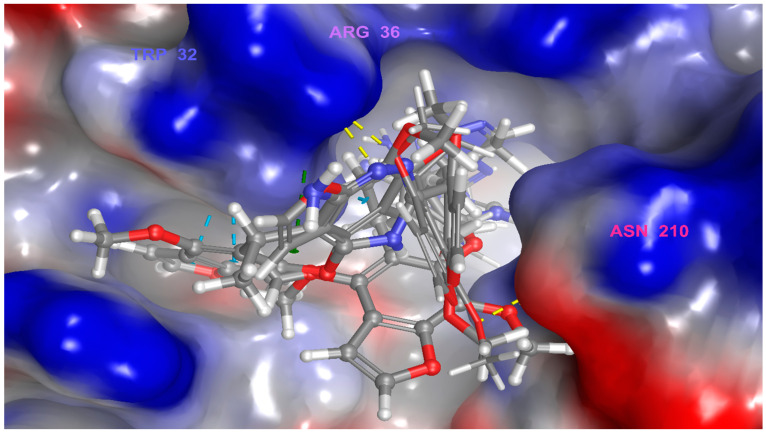
The best docked molecules are superimposed on the active site region of 1HNJ protein and active residues are visualized using Maestro.

The FabH active site generally contains a catalytic triad tunnel involving Cys112, His244, and Asn274. A change in these amino acid resides may inhibit or even stop an enzyme’s catalytic activity [57]. The direct outcome of this would be that fatty acid biosynthesis cannot carry on efficiently as the energy equipping the organism would not be enough, so the components of all cell membranes could not be formed, and antimicrobial activity would be revealed [58]. Subsequently, we carried out molecular docking studies of the prepared compounds with the crystal structure of *E. coli* FabH (entry 1HNJ in the Protein Data Bank) to discover their binding mode. From recent and previous scientific studies [59,60,61,62,63,64,65,66,67], we know that the hydroxyl (OH) group contributes greater affinity in the interaction of the receptor and the ligand as compared to the methoxy (OCH_3_) group by forming a hydrogen bond with the amino acid of the protein molecule, and the greater extent of hydrogen bonding leads to better interaction. In this study, molecular docking can offer worthwhile information on the action mechanism of our compounds. The docking results are shown in Table 3 and Figure 2, Figure 3 and Figure 4. In silico studies discovered that most of the prepared molecules had a good binding free energy (kcal/mol) for the target protein, ranging from −38.8 to −49.84 kcal/mol Table 3. Moreover, the changes in MM-GBSA accorded well with the MIC values obtained for most of the compounds—specifically, compounds (**20b**) and (**21b**), with good activity, exhibited very low MM-GBSA values of −42.40 and −49.84 kcal/mol, respectively. The observations from the biological assay data and the molecular docking results ability suggest that the antibacterial activity of these compounds is derived from the reaction between the compounds and the enzyme FabH.

## 3. Materials and Methods

Through cooperation with other researchers, a research plan was made for the synthesis of new heterocyclic compounds. These new compounds were planned to study their antimicrobial activity, and the plan was successfully implemented.

### 3.1. General Information

All the melting points were assessed on an Electrothermal IA 9100 series digital melting point apparatus (Shimadzu, Tokyo, Japan). Elemental analyses were performed on Vario EL (Elementar, Langenselbold, Germany). Microanalytical data were processed at the microanalytical center of the Faculty of Science at Cairo University and National Research Centre. The IR spectra (KBr disc) were recorded using a Perkin-Elmer 1650 spectrometer (Waltham, MA, USA). NMR spectra were determined using JEOL 270 MHz and JEOL JMS-AX 500 MHz (JEOL, Tokyo, Japan) spectrometers with Me4Si as an internal standard. Mass spectra were recorded on an EI Ms-QP 1000 EX instrument (Shimadzu, Tokyo, Japan) at 70 eV. Biological evaluations were performed by the antimicrobial unit of Department of Chemistry of Natural and Microbial Products (National Research Centre, Giza 12622, Egypt). All starting materials and solvents were purchased from Sigma-Aldrich (St. Louis, MO, USA).

### 3.2. Synthesis of 7-imino-(4-methoxy or 4, 9-dimethoxy)-5-methyl-7H-furo[3,2-g] chromene-6-carbonitrile (***3a,b***)

Method A. General Procedure [36]: To a stirred solution of visnaginone **2a** (2.06 g, 0.01 mol) or khellinone (**2b**) (2.36 g, 0.01 mol) and malononitrile (0.66 g, 0.01 mol) in absolute ethanol (50 mL) was added triethylamine (1 mL, 0.01 mol). The mixture was refluxed for 3–4 h (TLC) and then allowed to cool to room temperature. The final formed precipitate was isolated via filtration and washed with ethanol to get s pure product, then recrystallized from the proper solvent to give (**3a**) and (**3b**)**.**

Method B. To a stirred solution of visnaginone (**2a**) (2.06 g, 0.01 mol) or khellinone (**2b**) (2.36 g, 0.01 mol) in ethanolic sodium ethoxide solution (0.5 g, 0.02-atom of sodium 35 mL of ethanol), malononitrile (0.66 g, 0.01 mol) was added and the mixture was heated under reflux for 2–4 h and checked by TLC. After cooling, the final solid product was collected and recrystallized from the proper solvent to give (**3a**) and (**3b**), respectively.

### 3.3. Synthesis of 7-imino-4-methoxy-5-methyl-7H-furo[3,2-g]chromene-6-carbonitrile (***3a***)

The compound was obtained from the reaction of visnaginone (**2a**) (2.06 g, 0.01 mol) and malononitrile (0.66 g, 0.01 mol), as yellowish crystals, crystallized from dioxane (82%), melting point (M.p.): 208–210 °C. IR (ν, cm^−1^) KBr: 3300 (NH), 3055 (CH-aryl), 2965 (CH-aliph), 2240 (CN), 1630 (C=N), 1590 (C=C). ^1^H NMR (DMSO-d_6_, ppm) δ 2.35 (s, 3H, CH_3_), 3.80 (s, 3H, OCH_3_), 6.80 (d, 1H, *J* = 2.35 Hz, furan), 7.10 (s, 1H, benzene), 7.30 (d, 1H, *J* = 2.38 Hz, furan), 9.50 (s, 1H, NH, D_2_O exchangeable); ^13^C NMR (DMSO-d_6_) δ 18.5, 58.2 (2C, CH_3_, OCH_3_), 90.3 (1C, CH), 91.5, 116.7 (2C, C-CN), 103.1, 106.4, 110.2, 145.5, 150.7, 154.6, 155.2, 157.4, 161.2 (9C, Ar-C); MS (70 eV, %) m/z 254 (M^+^, 100%); Anal. Calc. (Found) for C_14_H_10_N_2_O_3_ (254.25): C, 66.14 (66.22); H, 3.96 (3.90); N, 11.02 (11.12).

### 3.4. Synthesis of 7-imino-4,9-dimethoxy-5-methyl-7H-furo[3,2-g]chromene-6-carbonitrile (***3b***) 

The compound was obtained from the reaction of khellinone (**2b**) (2.36 g, 0.01mol) and malononitrile (0.66 g, 0.01 mol), as yellow crystals, crystallized from methanol (80%), M.p.: 220–222 °C. IR (ν, cm^−1^) KBr: 3310 (NH), 3060 (CH-aryl), 2970 (CH-aliph), 2235 (CN), 1634 (C=N), 1595 (C=C). ^1^H NMR (DMSO-d_6_, ppm) δ 2.30 (s, 3H, CH_3_), 3.85 (s, 6H, 2OCH_3_), 6.85 (d, 1H, J = 2.34 Hz, furan),7.05 (d, 1H, J = 2.37 Hz, furan), 9.60 (s, 1H, NH, D_2_O exchangeable); ^13^C NMR (DMSO-d_6_) δ 18.6 (1C, CH_3_), 59.5 (2C, 2OCH_3_), 91.8, 116.9 (2C, C-CN), 103.5, 106.2, 113.1, 123.5, 145.1, 146.5, 148.4, 150.3, 157.2, 161.5 (10 C, Ar-C); MS (70 eV, %) m/z 284 (M^+^, 98%); Anal. Calc. (Found) for C_15_H_12_N_2_O_4_ (284.27): C, 63.38 (63.45); H, 4.26 (4.35); N, 9.85 (9.77).

### 3.5. Synthesis of 7-amino-(4-methoxy or 4,9-dimethoxy) -5-methyl-6,7-dihydro-5H-furo[3,2-g] chromene-6-carbonitrile (***4a,b***)

General procedure [36]: To a solution of (**3a**) (2.54 g, 0.01 mol) or (**3b**) (2.84 g, 0.01 mol) in methanol (50 mL) was added sodium borohydride (0.38 g, 0.01 mol) at 0 °C. The reaction mixture was stirred for 1–2 h, under control (TLC). The reaction mixture was poured into water and the precipitated solid was filtered, washed with water, and dried and crystallized from the proper solvent to give (**4a**) and (**4b**), respectively.

### 3.6. Synthesis of 7-amino-4-methoxy-5-methyl-6, 7-dihydro-5H-furo[3,2-g]chromene-6- carbonitrile (***4a***)

The compound was obtained from the reaction of (**3a**) (2.54 g, 0.01mol) and sodium borohydride (0.38 g, 0.01 mol), as white crystals, crystallized from ethanol (79%), M.p.: 240–242 °C. IR (ν, cm^−1^) KBr: broad 3416 (NH_2_), 3052 (CH-aryl), 2970 (CH-aliph), 2244 (CN), 1585 (C=C). ^1^H NMR (DMSO-d_6_, ppm) δ 1.45 (d, 3H, J = 6.80 Hz, CH_3_), 3.05 (m, 1H, CH, pyran ring), 3.15 (t, 1H, J = 6.85 Hz, CH_,_ pyran ring), 3.81 (s, 3H, OCH_3_), 5.10 (d, 1H, J = 6.88 Hz, CH_,_ pyran ring), 6.88 (d, 1H, J = 2.31 Hz, furan), 7.15 (s, 1H, benzene), 7.37 (d, 1H, J = 2.32 Hz, furan), 8.40 (s, 2H, NH_2_, D_2_O exchangeable); ^13^C NMR (DMSO-d_6_) δ 20.1 (1C, CH_3_), 21.3, 44.5, (2C, CH, pyran ring), 60.1 (1C, OCH_3_), 81.2 (1C, CH-NH_2_), 90.7 (1C, CH, benzene), 120.2 (1C, CN), 102.8, 105.1, 106.7, 145.9, 152.3, 154.8, 156.9 (7C, Ar-C); MS (70 eV, %) m/z 258 (M^+^, 100%); Anal. Calc. (Found) for C_14_H_14_N_2_O_3_ (258.28): C, 65.11 (65.20); H, 5.46 (5.52); N, 10.85 (10.77).

### 3.7. Synthesis of 7-amino-4,9-dimethoxy-5-methyl-6,7-dihydro-5H-furo[3,2-g]chromene-6- carbonitrile (***4b***)

The compound was obtained from the reaction of **3b** (2.84 g, 0.01mol) and sodium borohydride (0.38 g, 0.01 mol), as yellowish crystals, crystallized from benzene (77%), M.p.: 260–262 °C. IR (*ν*, cm^−1^) KBr: broad 3420 (NH_2_), 3057 (CH-aryl), 2962 (CH-aliph), 2241 (CN), 1582 (C=C). ^1^H NMR (DMSO-*d*_6_, ppm) *δ* 1.40 (d, 3H, *J* = 6.77 Hz, CH_3_), 3.08 (m, 1H, CH_,_ pyran ring), 3.11 (t, 1H, *J* = 6.87 Hz, CH_,_ pyran ring), 3.90 (s, 6H, 2OCH_3_), 5.15 (d, 1H, *J* = 6.81 Hz, CH_,_ pyran ring), 6.84 (d, 1H, *J* = 2.37 Hz, furan), 7.40 (d, 1H, *J* = 2.39 Hz, furan), 8.45 (s, 2H, NH_2_, D_2_O exchangeable); ^13^C NMR (DMSO-*d*_6_) *δ* 20.5 (1C, CH_3_), 21.7, 44.8, (2C, CH, pyran ring), 60.6 (2C, 2OCH_3_), 82.4 (1C, CH-NH_2_), 122.1 (1C, CN), 105.1, 105.7, 110.2, 127.3, 145.1, 145.8, 146.5, 146.9 (8C, Ar-C); MS (70 eV, %) *m*/*z* 288 (M^+^, 95%); Anal. Calc. (Found) for C_15_H_16_N_2_O_4_ (288.30): C, 62.49 (62.55); H, 5.59 (5.66); N, 9.72 (9.65).

### 3.8. Synthesis of (6-methoxy or 6, 10-dimethoxy) -5-methyl-4a, 11a-dihydro-5H-furo[3′,2′: 6,7] chromeno[2,3-d] pyrimidin-4-amine (***5a,b***)

General procedure: Method A. A mixture of (**4a**) (2.58 g, 0.01 mol) or (**4b**) (2.88 g, 0.01 mol) and formamide (30 mL) was refluxed for 4–6 h under control (TLC). After cooling, the yellowish crystals were filtered off and washed with cold water and methanol, then recrystallized from the proper solvent to give (**5a**) and (**5b**), respectively.

Method B. A stream of NH_3_ gas was passed through (**17a**) (3.14 g, 0.01 mol) or (**17b**) (3.44 g, 0.01 mol) in a dioxane solution at room temperature for 2–4 h under control (TLC). The mixture was left in the refrigerator overnight, and the solid product that formed upon cooling was collected by filtration to give (**5a**) and (**5b**), respectively.

### 3.9. Synthesis of 6-methoxy-5-methyl-4a, 11a-dihydro-5H-furo[3′,2′: 6,7]chromeno[2,3-d] pyrimidin-4-amine (***5a***)

The compound was obtained from the reaction of (**4a**) (2.58 g, 0.01 mol) and formamide (30 mL), as yellowish crystals, crystallized from methanol (75%), M.p. > 350 °C. IR (ν, cm^−1^) KBr: broad 3425 (NH_2_), 3057 (CH-aryl), 2973 (CH-aliph), 1628 (C=N), 1588 (C=C). ^1^H NMR (DMSO-d_6_, ppm) δ 1.38 (d, 3H, *J* = 6.75 Hz, CH_3_), 3.10 (m, 1H, CH_,_ pyran ring), 3.20 (t, 1H, *J* = 6.80 Hz, CH_,_ pyran ring), 3.85 (s, 3H, OCH_3_), 5.05 (d, 1H, *J* = 6.79 Hz, CH_,_ pyran ring), 6.45 (s, 2H, NH_2_, D_2_O exchangeable), 6.75 (d, 1H, *J* = 2.35 Hz, furan), 7.18 (s, 1H, benzene), 7.40 (d, 1H, *J* = 2.34 Hz, furan), 8.08 (s, 1H, CH_,_ pyrimidine ring); ^13^C NMR (DMSO-d_6_) δ 19.5 (1C, CH_3_), 23.5, 46.2, (2C, CH, pyran ring), 60.4 (1C, OCH_3_), 85.3 (1C, CH, pyran ring), 90.5 (1C, CH, benzene), 104.2, 105.5, 106.4, 147.2, 152.3, 154.1, 155.9, 157.4, 158.2 (9C, Ar-C); MS (70 eV, %) m/z 285 (M^+^, 100%); Anal. Calc. (Found) for C_15_H_15_N_3_O_3_ (285.30): C, 63.15 (63.22); H, 5.30 (5.39); N, 14.73 (14.66).

### 3.10. Synthesis of 6, 10-dimethoxy-5-methyl-4a, 11a-dihydro-5H-furo[3′,2′: 6,7]chromeno[2,3-d] pyrimidin-4-amine (***5b***)

The compound was obtained from the reaction of (**4b**) (2.88 g, 0.01 mol) and formamide (30 mL), as yellow crystals, crystallized from ethanol (73%), M.p. > 350 °C. IR (*ν*, cm^−1^) KBr: broad 3422 (NH_2_), 3059 (CH-aryl), 2977 (CH-aliph), 1629 (C=N), 1584 (C=C). ^1^H NMR (DMSO-*d*_6_, ppm) *δ* 1.35 (d, 3H, *J* = 6.73 Hz, CH_3_), 3.20 (m, 1H, CH_,_ pyran ring), 3.30 (t, 1H, *J* = 6.77 Hz, CH_,_ pyran ring), 3.91 (s, 6H, 2OCH_3_), 5.10 (d, 1H, *J* = 6.81 Hz, CH_,_ pyran ring), 6.50 (s, 2H, NH_2_, D_2_O exchangeable), 6.80 (d, 1H, *J* = 2.37 Hz, furan), 7.45 (d, 1H, *J* = 2.38 Hz, furan), 8.11 (s, 1H, CH_,_ pyrimidine ring); ^13^C NMR (DMSO-*d*_6_) *δ* 19.8 (1C, CH_3_), 24.2, 47.6, (2C, CH, pyran ring), 60.9 (2C, 2OCH_3_), 86.1 (1C, CH, pyran ring), 105.1, 106.3, 110.2, 127.4, 144.7, 145.5, 146.5, 147.1, 157.7, 158.5 (10 C, Ar-C); MS (70 eV, %) *m*/*z* 315 (M^+^, 100%); Anal. Calc. (Found) for C_16_H_17_N_3_O_4_ (315.33): C, 60.94 (60.88); H, 5.43 (5.50); N, 13.33 (13.40).

### 3.11. Synthesis of (6-methoxy or 6, 10-dimethoxy)-5-methyl-3, 5-dihydro-4H-furo[3′,2′: 6,7] chromeno[2,3-d]pyrimidin-4-one (***6a,b***)

General procedure: Method A. A solution of (**4a**) (2.58 g, 0.01 mol) or (**4b**) (2.88 g, 0.01 mol) and formic acid (25 mL) was heated under reflux for 7–10 h under control (TLC). The reaction solution was allowed to cool to room temperature and poured into water. The formed solid precipitate was collected by filtration, washed with ethanol, dried, and crystallized from the proper solvent to give (**6a**) and (**6b**). 

Method B. A mix of (**4a**) (2.58 g, 0.01 mol) or (**4b**) (2.88 g, 0.01 mol) and formic acid (10 mL) in formamide (35 mL) was refluxed for 5–8 h. After cooling, the solution was poured into cold water. The solid precipitate that formed was collected by filtration, washed with cold water/ethanol, and recrystallized from the proper solvent to give (**6a**) and (**6b**), respectively.

### 3.12. Synthesis of 6-methoxy-5-methyl-3, 5-dihydro-4H-furo[3′,2′: 6, 7]chromeno[2,3-d] pyrimidin-4-one (***6a***)

The compound was obtained from the reaction of (**4a**) (2.58 g, 0.01 mol) and formic acid (25 mL), as brownish crystals, crystallized from dioxane (81%), M.p. > 350 °C. IR (*ν*, cm^−1^) KBr: 3295 (br. NH), 3063 (CH-aryl), 2962 (CH-aliph), 1680 (CO), 1630 (C=N), 1582 (C=C). ^1^H NMR (DMSO-*d*_6_, ppm) *δ* 1.48 (d, 3H, *J* = 6.79 Hz, CH_3_), 3.65 (q, 1H, *J* = 6.76 Hz, CH_,_ pyran ring), 3.92 (s, 3H, OCH_3_), 6.81 (d, 1H, *J* = 2.32 Hz, furan), 7.07 (s, 1H, benzene), 7.45 (d, 1H, *J* = 2.37 Hz, furan), 8.12 (s, 1H, CH_,_ pyrimidine ring), 10.70 (s, 1H, NH, D_2_O exchangeable); ^13^C NMR (DMSO-*d*_6_) *δ* 19.4, (1C, CH, pyran ring), 22.1 (1C, CH_3_), 60.7 (1C, OCH_3_), 90.8 (1C, CH, benzene), 105.8, 106.3, 107.5, 109.6, 146.4, 150.7, 151.2, 153.8, 156.1, 160.5 (10C, Ar-C), 163.4 (1C, C=O); MS (70 eV, %) *m*/*z* 284 (M^+^, 100%); Anal. Calc. (Found) for C_15_H_12_N_2_O_4_ (284.27): C, 63.38 (63.45); H, 4.26 (4.35); N, 9.85 (9.77).

### 3.13. Synthesis of 6,10-dimethoxy-5-methyl-3, 5-dihydro-4H-furo[3′,2′: 6,7]chromeno[2,3-d] pyrimidin-4-one (***6b***)

The compound was obtained from the reaction of (**4b**) (2.88 g, 0.01 mol) and formic acid (25 mL), as yellowish crystals, crystallized from methanol (80%), M.p. > 350 °C. IR (*ν*, cm^−1^) KBr: 3290 (broad NH), 3060 (CH-aryl), 2959 (CH-aliph), 1682 (CO), 1633 (C=N), 1588 (C=C). ^1^H NMR (DMSO-*d*_6_, ppm) *δ* 1.50 (d, 3H, *J* = 6.80 Hz, CH_3_), 3.70 (q, 1H, *J* = 6.78 Hz, CH_,_ pyran ring), 3.95 (s, 6H, 2OCH_3_), 6.84 (d, 1H, *J* = 2.35 Hz, furan), 7.50 (d, 1H, *J* = 2.34 Hz, furan), 8.14 (s, 1H, CH_,_ pyrimidine ring), 10.75 (s, 1H, NH, D_2_O exchangeable); ^13^C NMR (DMSO-*d*_6_) *δ* 19.7, (1C, CH, pyran ring), 22.5 (1C, CH_3_), 60.9 (2C, 2OCH_3_), 106.1, 107.4, 110.6, 113.1, 124.3, 139.1, 145.8, 146.5, 146.9, 150.5, 161.8 (11C, Ar-C), 163.9 (1C, C=O); MS (70 eV, %) *m*/*z* 314 (M^+^, 100%); Anal. Calc. (Found) for C_16_H_14_N_2_O_5_ (314.30): C, 61.14 (61.22); H, 4.49 (4.55); N, 8.91 (8.84).

### 3.14. Synthesis of N-(6-cyano-(4-methoxy or 4, 9-dimethoxy)-5-methyl-5H-furo[3,2-g] chromen-7-yl) acetamide (***7a,b***)

General procedure: A mix of (**4a**) (2.58 g, 0.01 mol) and (**4b**) (2.88 g, 0.01 mol) was refluxed in acetic anhydride (30 mL) for 3–5 h, and then allowed to cool to room temperature and poured into cold water (50 mL). The solid product that formed was collected by filtration and washed with cold water. The final products were recrystallized from the proper solvent to give (**7a**) and (**7b**).

### 3.15. Synthesis of N-(6-cyano-4-methoxy-5-methyl-5H-furo[3,2-g]chromen-7-yl)acetamide (***7a***)

The compound was obtained from the reaction of (**4a**) (2.58 g, 0.01mol) and acetic anhydride (30 mL) as yellowish crystals, crystallized from ethanol (90%), M.p.: 318–320 °C. IR (*ν*, cm^−1^) KBr: 3300 (br. NH), 3057 (CH-aryl), 2962 (CH-aliph), 2225 (CN), 1691 (C=O), 1583 (C=C). ^1^H NMR (DMSO-*d*_6_, ppm) *δ* 1.30 (d, 3H, *J* = 6.83 Hz, CH_3_), 1.90 (s, 3H, CH_3_), 3.63 (q, 1H, *J* = 6.84 Hz, CH_,_ pyran ring), 3.87 (s, 3H, OCH_3_), 6.79 (d, 1H, *J* = 2.37 Hz, furan), 7.07 (s, 1H, benzene), 7.42 (d, 1H, *J* = 2.38 Hz, furan), 9.35 (s, 1H, NH, D_2_O exchangeable); ^13^C NMR (DMSO-*d*_6_) *δ* 19.6 (1C, CH, pyran ring), 22.7, 24.4 (2C, 2CH_3_), 60.2 (1C, OCH_3_), 68.1 (1C, C-CN, pyran ring), 118.6 (1C, CN), 90.1 (1C, CH, benzene), 104.5, 105.4, 108.8, 146.1, 150.5, 154.2, 155.1, 162.4 (8C, Ar-C), 168.5 (1C,C=O); MS (70 eV, %) *m*/*z* 298 (M^+^, 100%); Anal. Calc. (Found) for C_16_H_14_N_2_O_4_ (298.30): C, 64.42 (64.50); H, 4.73 (4.65); N, 9.39 (9.32).

### 3.16. Synthesis of N-(6-cyano-4,9-dimethoxy-5-methyl-5H-furo[3,2-g]chromen-7-yl)acetamide (***7b***)

The compound was obtained from the reaction of (**4b**) (2.88 g, 0.01mol) and acetic anhydride (30 mL) as yellow crystals, crystallized from methanol (85%), M.p.: 332–334 °C. IR (*ν*, cm^−1^) KBr: 3310 (br. NH), 3058 (CH-aryl), 2966 (CH-aliph), 2223 (CN), 1688 (C=O), 1580 (C=C). ^1^H NMR (DMSO-*d*_6_, ppm) *δ* 1.28 (d, 3H, *J* = 6.87 Hz, CH_3_), 1.84 (s, 3H, CH_3_), 3.59 (q, 1H, *J* = 6.78 Hz, CH_,_ pyran ring), 3.90 (s, 6H, 2OCH_3_), 6.81 (d, 1H, *J* = 2.38 Hz, furan), 7.47(d, 1H, *J* = 2.40 Hz, furan), 9.30 (s, 1H, NH, D_2_O exchangeable); ^13^C NMR (DMSO-*d*_6_) *δ* 19.3 (1C, CH, pyran ring), 22.1, 24.8 (2C, 2CH_3_), 61.3 (2C, 2OCH_3_), 68.5 (1C, C-CN, pyran ring), 118.8 (1C, CN), 106.2, 110.5, 112.7, 124.2, 139.6, 145.9, 146.4, 147.1, 163.7 (9C, Ar-C), 168.1 (1C,C=O); MS (70 eV, %) *m*/*z* 328 (M^+^, 100%); Anal. Calc. (Found) for C_17_H_16_N_2_O_5_ (328.32): C, 62.19 (62.27); H, 4.91 (4.84); N, 8.53 (8.60).

### 3.17. Synthesis of (6-methoxy or 6,10-dimethoxy)-2, 5-dimethyl-5H-furo [3′,2′: 6,7]chromeno [2,3-d]pyrimidin-4-ol (***8a,b***)

General procedure: Method A. A mixture of (**4a**) (2.58 g, 0.01 mol) and (**4b**) (2.88 g, 0.01 mol) in an acetic anhydride/pyridine mixture (30 mL:15 mL) was heated in a water bath for 10–12 h under control (TLC), allowed to cool, and poured into 30 mL of acidified cold water. The solid precipitate that formed was collected via filtration and washed with cold water. The precipitate products were recrystallized from the proper solvent to give (**8a**) and (**8b**).

Method B. A solution of (**7a**) (2.98 g, 0.01 mol) or (**7b**) (3.28 g, 0.01 mol) in absolute ethanol (25 mL) with pyridine (5 mL) was heated and refluxed on water bath for 6–9 h under control (TLC), after cooling the solid precipitate was collected via filtration, washed with water, dried and recrystallized from appropriate solvent to give (**8a**) and (**8b**).

### 3.18. Synthesis of 6-methoxy-2,5-dimethyl-5H-furo[3′,2′:6,7]chromeno[2,3-d]pyrimidin-4-ol (***8a***)

The compound was obtained from the reaction of (**4a**) (2.58 g, 0.01 mol) and acetic anhydride/pyridine as yellow crystals, crystallized from toluene (84%), M.p. > 350 °C. IR (*ν*, cm^−1^) KBr: 3412 (br. OH), 3071 (CH-aryl), 2960 (CH-aliph), 1632 (C=N), 1586 (C=C). ^1^H NMR (DMSO-*d*_6_, ppm) *δ* 1.50 (d, 3H, *J* = 6.78 Hz, CH_3_), 2.08 (s, 3H, CH_3_), 4.10 (q, 1H, *J* = 6.81 Hz, CH_,_ pyran ring), 3.82 (s, 3H, OCH_3_), 6.80 (d, 1H, *J* = 2.39 Hz, furan), 7.01 (s, 1H, benzene), 7.55 (d, 1H, *J* = 2.35 Hz, furan), 12.10 (s, 1H, OH, D_2_O exchangeable); ^13^C NMR (DMSO-*d*_6_) *δ* 22.1, 24.5 (2C, 2CH_3_), 24.9 (1C, CH, pyran ring), 60.4 (1C, OCH_3_), 93.2 (1C, CH, benzene), 105.6, 107.1, 112.7, 119.3, 146.4, 150.2, 153.8, 154.7, 156.1, 164.5, 169.8 (11C, Ar-C); MS (70 eV, %) *m*/*z* 298 (M^+^, 100%); Anal. Calc. (Found) for C_16_H_14_N_2_O_4_ (298.30): C, 64.42 (64.35); H, 4.73 (4.80); N, 9.39 (9.46).

### 3.19. Synthesis of 6, 10-dimethoxy-2, 5-dimethyl-5H-furo[3′,2′: 6,7]chromeno[2,3-d] pyrimidin-4-ol (***8b***)

The compound was obtained from the reaction of (**4b**) (2.88 g, 0.01 mol) and acetic anhydride/pyridine as yellowish crystals, crystallized from benzene (82%), M.p. > 350 °C. IR (*ν*, cm^−1^) KBr: 3408 (br. OH), 3073 (CH-aryl), 2962 (CH-aliph), 1636 (C=N), 1588 (C=C). ^1^H NMR (DMSO-*d*_6_, ppm) *δ* 1.58 (d, 3H, *J* = 6.74 Hz, CH_3_), 2.13 (s, 3H, CH_3_), 4.18 (q, 1H, *J* = 6.82 Hz, CH_,_ pyran ring), 3.94 (s, 6H, 2OCH_3_), 6.82 (d, 1H, *J* = 2.41 Hz, furan), 7.57 (d, 1H, *J* = 2.40 Hz, furan), 12.15 (s, 1H, OH, D_2_O exchangeable); ^13^C NMR (DMSO-*d*_6_) *δ* 22.6, 23.8 (2C, 2CH_3_), 24.7 (1C, CH, pyran ring), 62.3 (2C, 2OCH_3_), 106.5, 115.2, 115.8, 119.5, 128.5, 136.7, 144.9, 146.3, 146.8, 154.5, 165.6, 107.1 (12C, Ar-C); MS (70 eV, %) *m*/*z* 328 (M^+^, 100%); Anal. Calc. (Found) for C_17_H_16_N_2_O_5_ (328.32): C, 62.19 (62.27); H, 4.91 (4.85); N, 8.53 (8.60).

### 3.20. Synthesis of (7-methoxy or 7,11-dimethoxy)-2,6-dimethyl-1,6-dihydrofuro[3′,2′:6,7] chromeno[2,3-e][1,2,4]triazepin-5-amine (***9a,b***)

General procedure: A mix of (**7a**) (2.98 g, 0.01 mol) and (**7b**) (3.28 g, 0.01 mmol) and hydrazine hydrate (5 mL) in ethanol (40 mL) containing (0.1 mL) of piperidine was refluxed for 4–7 h under control (TLC). The reaction solution was concentrated under reduced pressure and the residue was triturated through methanol. The formed solid product was filtered, washed with methanol, and recrystallized from the appropriate solvent to give (**9a**) and (**9b**)**,** respectively.

### 3.21. Synthesis of 7-methoxy-2,6-dimethyl-1,6-dihydrofuro[3′,2′:6,7]chromeno[2,3-e][1,2,4] triazepin-5-amine (***9a***)

The compound was obtained from the reaction of (**7a**) (2.98 g, 0.01 mol) and hydrazine hydrate as yellow crystals, crystallized from DMF (78%), M.p. >350 °C. IR (*ν*, cm^−1^) KBr: broad 3420–3395 (NH_2_), 3305 (br. NH), 3050 (CH-aryl), 2960 (CH-aliph), 1630 (C=N), 1585 (C=C). ^1^H NMR (DMSO-*d*_6_, ppm) *δ* 1.28 (d, 3H, *J* = 6.90 Hz, CH_3_), 1.70 (s, 3H, CH_3_), 3.68 (q, 1H, *J* = 6.80 Hz, CH_,_ pyran ring), 3.82 (s, 3H, OCH_3_), 6.77 (d, 1H, *J* = 2.30 Hz, furan), 6.85 (s, 2H, NH_2_, D_2_O exchangeable), 7.01 (s, 1H, benzene), 7.50 (d, 1H, *J* = 2.31 Hz, furan), 10.10 (s, 1H, NH, D_2_O exchangeable); ^13^C NMR (DMSO-*d*_6_) *δ* 18.1 (1C, CH, pyran ring), 22.3, 24.1 (2C, 2CH_3_), 60.5 (1C, OCH_3_), 80.2 (1C, triazepin ring), 90.6 (1C, CH, benzene), 105.8, 106.7, 109.5, 146.4, 148.5, 148.6, 151.1, 153.8, 155.7, 160.2 (10 C, Ar-C); MS (70 eV, %) *m*/*z* 312 (M^+^, 90%); Anal. Calc. (Found) for C_16_H_16_N_4_O_3_ (312.33): C, 61.53 (61.60); H, 5.16 (5.21); N, 17.94 (17.88).

### 3.22. Synthesis of 7,11-dimethoxy-2,6-dimethyl-1,6-dihydrofuro[3′,2′:6,7]chromeno [2,3-e][1,2,4]triazepin-5-amine (***9b***)

The compound was obtained from the reaction of (**7b**) (3.28 g, 0.01 mmol) and hydrazine hydrate as yellowish crystals, crystallized from DMF (74%), M.p. > 350 °C. IR (*ν*, cm^−1^) KBr: broad 3415–3390 (NH_2_), 3301 (br. NH), 3052 (CH-aryl), 2963 (CH-aliph), 1634 (C=N), 1587 (C=C). ^1^H NMR (DMSO-*d*_6_, ppm) *δ* 1.29 (d, 3H, *J* = 6.92 Hz, CH_3_), 1.72 (s, 3H, CH_3_), 3.70 (q, 1H, *J* = 6.83 Hz, CH_,_ pyran ring), 3.88 (s, 6H, 2OCH_3_), 6.80 (d, 1H, *J* = 2.31 Hz, furan), 6.90 (s, 2H, NH_2_, D_2_O exchangeable), 7.55 (d, 1H, *J* = 2.36 Hz, furan), 10.20 (s, 1H, NH, D_2_O exchangeable); ^13^C NMR (DMSO-*d*_6_) *δ* 18.7 (1C, CH, pyran ring), 22.9, 24.4 (2C, 2CH_3_), 61.8 (2C, 2OCH_3_), 80.6 (1C, triazepin ring), 106.1, 110.4, 112.5, 124.3, 139.2, 145.6, 146.2, 146.9, 148.5, 148.7, 160.4 (11 C, Ar-C); MS (70 eV, %) *m*/*z* 342 (M^+^, 85%); Anal. Calc. (Found) for C_17_H_18_N_4_O_4_ (342.35): C, 59.64 (59.71); H, 5.30 (5.37); N, 16.37 (16.30).

### 3.23. Synthesis of 7-amino-(4-methoxy or 4, 9-dimethoxy)-5-methyl-5H-furo[3,2-g]chromene- 6-carboxamide (***10a,b***)

General procedure: Method A. Compound (**4a**) (2.58 g, 0.01 mol) or (**4b**) (2.88 g, 0.01 mol) was stirred in concentrated H_2_SO_4_ (30 mL) for 20–24 h at room temperature. The reaction mixture was poured dropwise over crushed ice. The solid product was filtered, washed with water, left to dry and recrystallized from the appropriate solvent to give (**10a**) or (**10b**), respectively.

Method B. Compound (**4a**) (2.58 g, 0.01 mol) or (**4b**) (2.88 g, 0.01 mol) was added dropwise with stirring to concentrated cold sulfuric acid at 20 °C (15 mL); so long as the temperature did not exceed 40 °C, the solution was stirred for a further 2 h at room temperature and poured into ice-cold water (20 mL). The reaction solution was left overnight in the refrigerator. The final solid precipitate was filtered off and recrystallized from the proper solvent to give (**10a**) or (**10b**).

### 3.24. Synthesis of 7-amino-4-methoxy-5-methyl-5H-furo[3,2-g]chromene-6-carboxamide (***10a***)

The compound was obtained from the reaction of (**4a**) (2.58 g, 0.01 mol) and concentrated cold sulfuric acid as brownish crystals, crystallized from methanol (73%), M.p.: 345–347 °C. IR (*ν*, cm^−1^) KBr: broad 3415, 3405 (2NH_2_), 3080 (CH-aryl), 2930 (CH-aliph), 1660 (C=O), 1581 (C=C). ^1^H NMR (DMSO-*d*_6_, ppm) *δ* 1.38 (d, 3H, *J* = 6.72 Hz, CH_3_), 3.65 (q, 1H, *J* = 6.75 Hz, CH_,_ pyran ring), 3.87 (s, 3H, OCH_3_), 6.74 (d, 1H, *J* = 2.35 Hz, furan), 6.86 (s, 2H, NH_2_, D_2_O exchangeable), 7.09 (s, 1H, benzene), 7.25 (s, 2H, 2NH_2_, D_2_O exchangeable), 7.74 (d, 1H, *J* = 2.42 Hz, furan); ^13^C NMR (DMSO-*d*_6_) *δ* 19.2 (1C, CH, pyran ring), 21.5 (1C, CH_3_), 60.2 (1C, OCH_3_), 84.5 (1C, pyran ring), 90.7 (1C, CH, benzene), 105.2, 106.6, 110.3, 146.7, 150.9, 153.5, 155.8, 159.2 (8C, Ar-C), 170.1(1C,C=O); MS (70 eV, %) *m*/*z* 274 (M^+^, 100%); Anal. Calc. (Found) for C_14_H_14_N_2_O_4_ (274.28): C, 61.31 (61.38); H, 5.15 (5.22); N, 10.21 (10.27).

### 3.25. Synthesis of 7-amino-4,9-dimethoxy-5-methyl-5H-furo[3,2-g]chromene-6-carboxamide (***10b***)

The compound was obtained from the reaction of (**4b**) (2.88 g, 0.01 mol) and conc. cold sulfuric acid as yellowish crystals, crystallized from ethanol (70%), M.p. > 350 °C. IR (*ν*, cm^−1^) KBr: broad 3410, 3402 (2NH_2_), 3085 (CH-aryl), 2935 (CH-aliph), 1665 (C=O), 1583 (C=C). ^1^H NMR (DMSO-*d*_6_, ppm) *δ* 1.40 (d, 3H, *J* = 6.71 Hz, CH_3_), 3.70 (q, 1H, *J* = 6.79 Hz, CH_,_ pyran ring), 3.92 (s, 6H, 2OCH_3_), 6.80 (d, 1H, *J* = 2.40 Hz, furan), 6.91 (s, 2H, NH_2_, D_2_O exchangeable), 7.30 (s, 2H, 2NH_2_, D_2_O exchangeable), 7.70 (d, 1H, *J* = 2.44 Hz, furan); ^13^C NMR (DMSO-*d*_6_) *δ* 18.9 (1C, CH, pyran ring), 21.7 (1C, CH_3_), 61.8 (2C, 2OCH_3_), 85.1 (1C, pyran ring), 106.4, 111.5, 113.7, 124.3, 138.5, 144.9, 146.4, 147.1, 158.7 (9C, Ar-C), 173.5 (1C,C=O); MS (70 eV, %) *m*/*z* 304 (M^+^, 92%); Anal. Calc. (Found) for C_15_H_16_N_2_O_5_ (304.30): C, 59.21 (59.30); H, 5.30 (5.39); N, 9.21 (9.14).

### 3.26. Synthesis of (6-methoxy or 6,10-dimethoxy)-5-methyl-2-phenyl-3,5-dihydro-4H-furo[3′,2′: 6,7]chromeno[2,3-d]pyrimidin-4-one (***11a,b***)

General procedure: A mix of compounds (**10a**) (2.74 g, 0.01 mol) and (**10b**) (3.04 g, 0.01 mol) and the suitable acid chloride (0.01 mol), namely benzoyl chloride (1.2 mL, 0.01 mol), was refluxed in acetic acid (25 mL) for 9–12 h under control (TLC). The reaction solution was allowed to cool, then poured onto ice-cold water. The solid precipitate was filtered, washed with water, and recrystallized from the proper solvent to give (**11a**) and (**11b**). 

### 3.27. Synthesis of 6-methoxy-5-methyl-2-phenyl-3,5-dihydro-4H-furo[3′,2′: 6,7]chromeno[2,3-d] pyrimidin-4-one (***11a***)

The compound was obtained from the reaction of (**10a**) (2.74 g, 0.01 mol) and benzoyl chloride as yellowish crystals, crystallized from dioxane (71%), M.p. > 350 °C. IR (*ν*, cm^−1^) KBr: broad 3270 (NH), 3084 (CH-aryl), 2935 (CH-aliph), 1686 (C=O), 1585 (C=C). ^1^H NMR (DMSO-*d*_6_, ppm) *δ* 1.40 (d, 3H, *J* = 6.80 Hz, CH_3_), 3.70 (q, 1H, *J* = 6.81 Hz, CH_,_ pyran ring), 3.90 (s, 3H, OCH_3_), 6.78 (d, 1H, *J* = 2.39 Hz, furan), 7.07 (s, 1H, benzene),7.35–7.72 (m, 5H, phenyl), 7.78 (d, 1H, *J* = 2.41 Hz, furan), 11.10 (s, 1H, NH_,_ D_2_O exchangeable); ^13^C NMR (DMSO-*d*_6_) *δ* 19.5 (1C, CH, pyran ring), 21.8 (1C, CH_3_), 60.7 (1C, OCH_3_), 90.8 (1C, CH, benzene), 105.4, 106.1, 108.1, 109.3, 128.1, 128.6, 130.4, 131.5, 146.2, 150.5, 153.1, 155.6, 158.7, 160.5 (16C, Ar-C), 165.2(1C,C=O); MS (70 eV, %) *m*/*z* 360 (M^+^, 100%); Anal. Calc. (Found) for C_21_H_16_N_2_O_4_ (360.37): C, 69.99 (69.90); H, 4.48 (4.41); N, 7.77 (7.84).

### 3.28. Synthesis of 6, 10-dimethoxy-5-methyl-2-phenyl-3, 5-dihydro-4H-furo[3′,2′: 6,7]chromeno [2,3-d]pyrimidin-4-one (***11b***)

The compound was obtained from the reaction of (**10b**) (3.04 g, 0.01 mol) and benzoyl chloride as brownish crystals, crystallized from THF (68%), M.p. > 350 °C. IR (*ν*, cm^−1^) KBr: broad 3275 (NH), 3080 (CH-aryl), 2932 (CH-aliph), 1682 (C=O), 1580 (C=C). ^1^H NMR (DMSO-*d*_6_, ppm) *δ* 1.41 (d, 3H, *J* = 6.82 Hz, CH_3_), 3.68 (q, 1H, *J* = 6.84 Hz, CH_,_ pyran ring), 3.94 (s, 6H, 2OCH_3_), 6.80 (d, 1H, *J* = 2.37 Hz, furan), 7.40–7.79 (m, 5H, phenyl), 7.85 (d, 1H, *J* = 2.34 Hz, furan), 11.22 (s, 1H, NH_,_ D_2_O exchangeable); ^13^C NMR (DMSO-*d*_6_) *δ* 19.2 (1C, CH, pyran ring), 22.1 (1C, CH_3_), 60.9 (2C, 2OCH_3_), 106.5, 107.8, 110.5,112.6, 123.5, 128.4, 128.9, 130.5, 131.9, 139.2, 145.4, 146.6, 146.9, 157.1, 160.8 (17C, Ar-C), 166.1 (1C,C=O); MS (70 eV, %) *m*/*z* 390 (M^+^, 94%); Anal. Calc. (Found) for C_22_H_18_N_2_O_5_ (390.39): C, 67.69 (67.75); H, 4.65 (4.71); N, 7.18 (7.10).

### 3.29. Synthesis of (6-methoxy or 6,10-dimethoxy)-5-methyl-1,4a,5,11a-tetrahydro-2H-furo[3′,2′: 6,7]chromeno [2,3-d]pyrimidine-2,4(3H)-dithione (***12a,b***)

General procedure: A solution of compound (**4a**) (2.58 g, 0.01 mol) or (**4b**) (2.88 g, 0.01 mol) and carbon disulfide (4 mL) in 40 mL of pyridine was heated and stirred under reflux on a water bath for 11–14 h with TLC. The solid product precipitated so formed was filtered off while hot and washed several times with ethanol. The final products were recrystallized from the suitable solvent to give (**12a**) or (**12b**).

### 3.30. Synthesis of 6-methoxy-5-methyl-1,4a,5,11a-tetrahydro-2H-furo[3′,2′:6,7]chromeno[2,3-d] pyrimidine-2,4(3H)-dithione (***12a***)

The compound was obtained from the reaction of (**4a**) (2.58 g, 0.01 mol) and carbon disulfide, as pale yellow crystals, crystallized from DMF (68%), M.p. > 350 °C. IR (*ν*, cm^−1^) KBr: IR (*ν*, cm^−1^) KBr: broad 3235-3210 (2NH), 3059 (CH-aryl), 2977 (CH-aliph), 1581 (C=C), 1300–1295 (2C=S), ^1^H NMR (DMSO-*d*_6_, ppm) *δ* 1.33 (d, 3H, *J* = 6.74 Hz, CH_3_), 3.08 (m, 1H, CH_,_ pyran ring), 3.12 (t, 1H, *J* = 6.75 Hz, CH_,_ pyran ring), 3.90 (s, 3H, OCH_3_), 5.20 (d, 1H, *J* = 6.77 Hz, CH_,_ pyran ring), 6.80 (d, 1H, *J* = 2.34 Hz, furan), 7.10 (s, 1H, benzene), 7.42 (d, 1H, *J* = 2.37 Hz, furan), 9.10, 12.02 (s, 2H, 2NH, D_2_O exchangeable); ^13^C NMR (DMSO-*d*_6_) *δ* 19.3 (1C, CH_3_), 32.5 (1C, CH, pyran ring), 60.8 (1C, OCH_3_), 70.2, 90.5 (2C, 2CH, pyran ring), 92.1 (1C, CH, benzene), 103.6, 105.4, 106.9, 146.5, 151.6, 154.1, 155.7 (7C, Ar-C), 180.1, 195.8 (2C, C=S); MS (70 eV, %) *m*/*z* 334 (M^+^, 92%); Anal. Calc. (Found) for C_15_H_14_N_2_O_3_S_2_ (334.41): C, 53.88 (53.80); H, 4.22 (4.30); N, 8.38 (8.45).

### 3.31. Synthesis of 6,10-dimethoxy-5-methyl-1,4a,5,11a-tetrahydro-2H-furo[3′,2′:6,7]chromeno [2, 3-d]pyrimidine-2,4(3H)-dithione (***12b***)

The compound was obtained from the reaction of (**4b**) (2.88 g, 0.01 mol) and carbon disulfide, as yellowish crystals, crystallized from dioxane (66%), M.p. > 350 °C. IR (*ν*, cm^−1^) KBr: IR (*ν*, cm^−1^) KBr: broad 3231–3207 (2NH), 3060 (CH-aryl), 2980 (CH-aliph), 1580 (C=C), 1302–1297 (2C=S), ^1^H NMR (DMSO-*d*_6_, ppm) *δ* 1.28 (d, 3H, *J* = 6.71 Hz, CH_3_), 3.12 (m, 1H, CH_,_ pyran ring), 3.20 (t, 1H, *J* = 6.76 Hz, CH_,_ pyran ring), 3.95 (s, 6H, 2OCH_3_), 5.30 (d, 1H, *J* = 6.78 Hz, CH_,_ pyran ring), 6.82 (d, 1H, *J* = 2.30 Hz, furan), 7.48 (d, 1H, *J* = 2.32 Hz, furan), 9.20, 12.15 (s, 2H, 2NH, D_2_O exchangeable); ^13^C NMR (DMSO-*d*_6_) *δ* 19.7 (1C, CH_3_), 33.1 (1C, CH, pyran ring), 61.8 (2C, 2OCH_3_), 70.5, 90.9 (2C, 2CH, pyran ring), 105.5, 106.3, 110.4, 127.1, 144.7, 145.6, 146.4, 146.8 (8C, Ar-C), 180.5, 196.1 (2C, C=S); MS (70 eV, %) *m*/*z* 364 (M^+^, 85%); Anal. Calc. (Found) for C_16_H_16_N_2_O_4_S_2_ (364.43): C, 52.73 (52.80); H, 4.43 (4.35); N, 7.69 (7.78).

### 3.32. Synthesis of N-(6-cyano-(4-methoxy or 4,9-dimethoxy)-5-methyl-6,7-dihydro-5H-furo[3, 2-g]chromen-7-yl)-2-phenylacetamide (***13a,b***)

General procedure: A mixture of compound (**4a**) (2.58 g, 0.01 mol) or (**4b**) (2.88 g, 0.01 mol) and 2-phenylacetyl chloride (1.55 g, 0.01 mol) in 35 mL of pyridine was heated and refluxed for 7–10 h with TLC. The reaction solution was allowed to cool at room temperature and then poured into acidified cold water. The final precipitate was filtered, washed with cold water, dried, and crystallized with the appropriate solvent to give (**13a**) or (**13b**).

### 3.33. Synthesis of N-(6-cyano-4-methoxy-5-methyl-6,7-dihydro-5H-furo[3,2-g]chromen-7-yl)- 2-phenyl- acetamide (***13a***)

The compound was obtained from the reaction of (**4a**) (2.58 g, 0.01mol) and 2-phenylacetyl chloride (1.55 g, 0.01mol), as brownish crystals, crystallized from *n*-hexane (90%), M.p.: 292–294 °C. IR (*ν*, cm^−1^) KBr: broad 3215 (NH), 3045 (CH-aryl), 2962 (CH-aliph), 2240 (CN), 1690 (C=O), 1582 (C=C). ^1^H NMR (DMSO-*d*_6_, ppm) *δ* 1.41 (d, 3H, *J* = 6.73 Hz, CH_3_), 3.21 (m, 1H, CH_,_ pyran ring), 3.28 (t, 1H, *J* = 6.79 Hz, CH_,_ pyran ring), 3.35 (s, 2H, CH_2_), 3.88 (s, 3H, OCH_3_), 5.35 (d, 1H, *J* = 6.83 Hz, CH_,_ pyran ring), 6.74 (d, 1H, *J* = 2.39 Hz, furan), 7.02–7.26 (s, 5H, benzene), 7.30 (s, 1H, benzene), 7.50 (d, 1H, *J* = 2.38 Hz, furan), 9.22 (s, 1H, NH, D_2_O exchangeable); ^13^C NMR (DMSO-*d*_6_) *δ* 19.4 (1C, CH_3_), 20.5, 38.7, (2C, CH, pyran ring), 39.5 (1C, CH_2_), 60.6 (1C, OCH_3_), 82.3 (1C, CH-pyran ring), 91.1 (1C, CH, benzene), 120.5 (1C, CN), 103.4, 105.3, 106.5, 127.4, 128.8, 129.1, 136.1, 146.7, 152.1, 153.9, 156.5 (13C, Ar-C), 170.8 (1C, C=O); MS (70 eV, %) *m*/*z* 376 (M^+^, 100%); Anal. Calc. (Found) for C_22_H_20_N_2_O_4_ (376.41): C, 70.20 (70.28); H, 5.36 (5.45); N, 7.44 (7.52).

### 3.34. Synthesis of N-(6-cyano-4, 9-dimethoxy-5-methyl-6,7-dihydro-5H-furo[3,2-g]chromen- 7-yl)-2-phenylacetamide (***13b***)

The compound was obtained from the reaction of (**4b**) (2.88 g, 0.01mol) and 2-phenylacetyl chloride (1.55 g, 0.01 mol), as yellowish crystals, crystallized from benzene (85%), M.p.: 305–307 °C. IR (*ν*, cm^−1^) KBr: broad 3220 (NH), 3050 (CH-aryl), 2966 (CH-aliph), 2244 (CN), 1692 (C=O), 1585 (C=C). ^1^H NMR (DMSO-*d*_6_, ppm) *δ* 1.38 (d, 3H, *J* = 6.72 Hz, CH_3_), 3.19 (m, 1H, CH_,_ pyran ring), 3.25 (t, 1H, *J* = 6.75 Hz, CH_,_ pyran ring), 3.33 (s, 2H, CH_2_), 3.95 (s, 6H, 2OCH_3_), 5.45 (d, 1H, *J* = 6.84 Hz, CH_,_ pyran ring), 6.71 (d, 1H, *J* = 2.45 Hz, furan), 7.05–7.30 (s, 5H, benzene), 7.55 (d, 1H, *J* = 2.44 Hz, furan), 9.30 (s, 1H, NH, D_2_O exchangeable); ^13^C NMR (DMSO-*d*_6_) *δ* 19.6 (1C, CH_3_), 20.8, 40.2, (2C, CH, pyran ring), 39.7(1C, CH_2_), 61.2 (2C, 2OCH_3_), 82.6 (1C, CH-pyran ring),120.7 (1C, CN), 106.1, 107.4, 110.2, 126.7, 127.5, 129.4, 129.8, 136.9, 145.1, 146.2, 146.5, 146.9 (14C, Ar-C), 171.3 (1C, C=O); MS (70 eV, %) *m*/*z* 406 (M^+^, 88%); Anal. Calc. (Found) for C_23_H_22_N_2_O_5_ (406.44): C, 67.97 (67.90); H, 5.46 (5.55); N, 6.89 (6.80).

### 3.35. Synthesis of 2-benzyl-(6-methoxy or 6,10-dimethoxy)-5-methyl-1,4a,5,11a-tetrahydro- 4H-furo[3′,2′: 6,7]chromeno [2,3-d]pyrimidin-4-one (***14a,b***)

General procedure: To a well-stirred cold mixture of compound (**13a**) (3.76 g, 0.01 mol) or (**13b**) (4.06 g, 0.01 mol) in 20 mL of (HCl: AcOH/1:1), a cold solution of H_2_O_2_ (20 mL) was added dropwise in an ice bath (0–5 °C), and the reaction solution was stirred for 6–8 h at room temperature. The solid that precipitated was collected by filtration, then redissolved in NaOH (30 mL 10%), heated under reflux for 30–60 min, and cooled. The result was acidified with HCl (25 mL) and the final solid product was collected and crystallized from the proper solvent to give (**14a**) or (**14b**).

### 3.36. Synthesis of 2-benzyl-6-methoxy-5-methyl-1,4a,5,11a-tetrahydro-4H-furo[3′,2′:6,7] chromeno[2,3-d]pyrimidin-4-one (***14a***)

The compound was obtained from the reaction of (**13a**) (3.76 g, 0.01 mol) and H_2_O_2,_ as yellowish crystals, crystallized from DMF (55%), M.p. > 350 °C. IR (*ν*, cm^−1^) KBr: broad 3290 (NH), 3075 (CH-aryl), 2970 (CH-aliph), 1677 (C=O), 1626 (C=N), 1585 (C=C). ^1^H NMR (DMSO-*d*_6_, ppm) *δ* 1.37 (d, 3H, *J* = 6.71 Hz, CH_3_), 3.15 (m, 1H, CH_,_ pyran ring), 3.31 (t, 1H, *J* = 6.74 Hz, CH_,_ pyran ring), 3.62 (s, 2H, CH_2_), 3.80 (s, 3H, OCH_3_), 5.22 (d, 1H, *J* = 6.79 Hz, CH_,_ pyran ring), 6.71 (d, 1H, *J* = 2.41 Hz, furan), 7.10 (s, 1H, benzene), 7.20–7.40 (s, 5H, benzene), 7.60 (d, 1H, *J* = 2.38 Hz, furan), 9.50 (s, 1H, NH, D_2_O exchangeable); ^13^C NMR (DMSO-*d*_6_) *δ* 19.8 (1C, CH_3_), 30.5 (1C, CH, pyran ring), 38.9 (1C, CH_2_), 60.2 (1C, OCH_3_), 63.5, 84.7 (1C, CH-pyran ring), 91.8 (1C, CH, benzene), 103.6, 105.5, 106.8, 126.1, 128.4, 129.3, 135.8, 146.4, 152.5, 154.2, 155.8, 157.4 (14C, Ar-C), 172.1 (1C, C=O); MS (70 eV, %) *m*/*z* 376 (M^+^, 77%); Anal. Calc. (Found) for C_22_H_20_N_2_O_4_ (376.41): C, 70.20 (70.12); H, 5.36 (5.28); N, 7.44 (7.57).

### 3.37. Synthesis of 2-benzyl-6,10-dimethoxy-5-methyl-1,4a,5,11a-tetrahydro-4H-furo[3′,2′:6, 7]chromeno[2,3-d]pyrimidin-4-one (***14b***)

The compound was obtained from the reaction of (**13b**) (4.06 g, 0.01 mol) and H_2_O_2,_ as yellow crystals, crystallized from dioxane (52%), M.p. > 350 °C. IR (*ν*, cm^−1^) KBr: broad 3285 (NH), 3070 (CH-aryl), 2972 (CH-aliph), 1675 (C=O), 1628 (C=N), 1582 (C=C). ^1^H NMR (DMSO-*d*_6_, ppm) *δ* 1.33 (d, 3H, *J* = 6.73 Hz, CH_3_), 3.20 (m, 1H, CH_,_ pyran ring), 3.35 (t, 1H, *J* = 6.71 Hz, CH_,_ pyran ring), 3.66 (s, 2H, CH_2_), 3.94 (s, 6H, 2OCH_3_), 5.30 (d, 1H, *J* = 6.83 Hz, CH_,_ pyran ring), 6.85 (d, 1H, *J* = 2.45 Hz, furan), 7.22–7.42 (s, 5H, benzene), 7.68 (d, 1H, *J* = 2.43 Hz, furan), 9.60 (s, 1H, NH, D_2_O exchangeable); ^13^C NMR (DMSO-*d*_6_) *δ* 18.9 (1C, CH_3_), 30.8 (1C, CH, pyran ring), 39.4 (1C, CH_2_), 61.1 (2C, 2OCH_3_), 63.8, 85.1 (1C, CH-pyran ring), 106.1, 106.7, 110.4, 125.9, 126.6, 128.8, 129.5, 135.9, 145.1, 146.5, 146.8, 147.2, 157.1 (15C, Ar-C), 172.5 (1C, C=O); MS (70 eV, %) *m*/*z* 406 (M^+^, 70%); Anal. Calc. (Found) for C_23_H_22_N_2_O_5_ (406.44): C, 67.97 (67.91); H, 5.46 (5.40); N, 6.89 (6.79).

### 3.38. Synthesis of 4-imino-(6-methoxy or 6,10-dimethoxy)-5-methyl-3-phenyl-1,3,4,4a,5,11a- hexahydro-2H-furo [3′,2′: 6,7]chromeno [2,3-d]pyrimidine-2-thione (***15a,b***) and 4-imino-(6- methoxy or 6,10-dimethoxy)-5-methyl-3-phenyl-1,3,4,4a,5,11a-hexahydro-2H-furo[3′,2′:6,7] chromeno[2,3-d]pyrimidin-2-one (***16a,b***)

General procedure: A solution of compound (**4a**) (2.58 g, 0.01 mol) or (**4b**) (2.88 g, 0.01 mol) and phenylisothiocyanate (1.60 mL, 0.01 mol) or phenylisocyanate (1.10 mL, 0.01 mol) in 40 mL of pyridine was heated and refluxed for 21–24 h under control (TLC). The mixture was cooled and poured into cold water, filtrated, washed several times with ethanol, and dried. The final product was recrystallized from the proper solvent to give (**15a**), (**15b**), (**16a**), or (**16b**), respectively.

### 3.39. Synthesis of 4-imino-6-methoxy-5-methyl-3-phenyl-1,3,4,4a,5,11a-hexahydro-2H-furo [3′,2′:6,7]chromeno[2,3-d]pyrimidine-2-thione (***15a***)

The compound was obtained from the reaction of (**4a**) (2.58 g, 0.01 mol) and phenylisothiocyanate as brownish crystals, crystallized from methanol (60%), M.p. > 350 °C. IR (*ν*, cm^−1^) KBr: broad 3300-3225 (2NH), 3071(CH-aryl), 2966 (CH-aliph), 1633 (C=N), 1585 (C=C), 1335 (C=S), ^1^H NMR (DMSO-*d*_6_, ppm) *δ* 1.32 (d, 3H, *J* = 6.74 Hz, CH_3_), 3.10 (m, 1H, CH_,_ pyran ring), 3.27 (t, 1H, *J* = 6.72 Hz, CH_,_ pyran ring), 3.78 (s, 3H, OCH_3_), 5.15 (d, 1H, *J* = 6.73 Hz, CH_,_ pyran ring), 6.74 (d, 1H, *J* = 2.38 Hz, furan), 7.14 (s, 1H, benzene), 7.35–7.58 (s, 5H, benzene ring), 7.70 (d, 1H, *J* = 2.36 Hz, furan), 9.10 (s, 1H, NH, D_2_O exchangeable), 9.60 (s, 1H, NH, D_2_O exchangeable); ^13^C NMR (DMSO-*d*_6_) *δ* 19.5 (1C, CH_3_), 30.1, 53.8 (2C, 2CH, pyran ring), 60.6 (1C, OCH_3_), 88.8 (1C, CH-pyran ring), 91.9 (1C, CH, benzene), 103.3, 105.2, 106.4, 128.1, 129.2, 131.4, 133.2, 146.1, 152.4, 154.1, 155.6, 157.1 (14C, Ar-C), 175.5 (1C, C=S); MS (70 eV, %) *m*/*z* 393 (M^+^, 80%); Anal. Calc. (Found) for C_21_H_19_N_3_O_3_S (393.46): C, 64.11 (64.20); H, 4.87 (4.80); N, 10.68 (10.61).

### 3.40. Synthesis of 4-imino-6,10-dimethoxy-5-methyl-3-phenyl-1,3,4,4a,5,11a-hexahydro-2H-furo [3′,2′:6,7]chromeno[2,3-d]pyrimidine-2-thione (***15b***)

The compound was obtained from the reaction of (**4b**) (2.88 g, 0.01 mol) and phenylisothiocyanate as yellowish crystals, crystallized from n-hexane (58%), M.p. > 350 °C. IR (*ν*, cm^−1^) KBr: broad 3305–3230 (2NH), 3074 (CH-aryl), 2968 (CH-aliph), 1631 (C=N), 1583 (C=C), 1332 (C=S), ^1^H NMR (DMSO-*d*_6_, ppm) *δ* 1.28 (d, 3H, *J* = 6.70 Hz, CH_3_), 3.08 (m, 1H, CH_,_ pyran ring), 3.24 (t, 1H, *J* = 6.77 Hz, CH_,_ pyran ring), 3.88 (s, 6H, 2OCH_3_), 5.23 (d, 1H, *J* = 6.78 Hz, CH_,_ pyran ring), 6.80 (d, 1H, *J* = 2.40 Hz, furan),7.38–7.61(s, 5H, benzene ring), 7.82 (d, 1H, *J* = 2.42 Hz, furan), 9.20 (s, 1H, NH, D_2_O exchangeable), 9.70 (s, 1H, NH, D_2_O exchangeable); ^13^C NMR (DMSO-*d*_6_) *δ* 20.1 (1C, CH_3_), 30.7, 54.2 (2C, 2CH, pyran ring), 60.9 (2C, 2OCH_3_), 89.5 (1C, CH-pyran ring), 105.9, 106.4, 110.2, 126.8, 128.6, 129.5, 131.7, 134.3, 145.1, 146.2, 146.7, 147.1, 157.3 (15C, Ar-C), 175.8 (1C, C=S); MS (70 eV, %) *m*/*z* 423 (M^+^, 75%); Anal. Calc. (Found) for C_22_H_21_N_3_O_4_S (423.49): C, 62.40 (62.50); H, 5.00 (5.10); N, 9.92 (9.83).

### 3.41. Synthesis of 4-imino-6-methoxy-5-methyl-3-phenyl-1,3,4,4a,5,11a-hexahydro-2H-furo [3′,2′:6,7]chromeno[2,3-d]pyrimidin-2-one (***16a***)

The compound was obtained from the reaction of (**4a**) (2.58 g, 0.01 mol) and phenylisocyanate as yellow crystals, crystallized from toluene (57%), M.p. > 350 °C. IR (*ν*, cm^−1^) KBr: broad 3290–3240 (2NH), 3080 (CH-aryl), 2971 (CH-aliph), 1688 (C=O), 1635 (C=N), 1590 (C=C), ^1^H NMR (DMSO-*d*_6_, ppm) *δ* 1.36 (d, 3H, *J* = 6.77 Hz, CH_3_), 3.08 (m, 1H, CH_,_ pyran ring), 3.30 (t, 1H, *J* = 6.79 Hz, CH_,_ pyran ring), 3.91 (s, 3H, OCH_3_), 5.25 (d, 1H, *J* = 6.78 Hz, CH_,_ pyran ring), 6.79 (d, 1H, *J* = 2.41 Hz, furan), 7.27 (s, 1H, benzene), 7.39–7.61 (s, 5H, phenyl ring), 7.68 (d, 1H, *J* = 2.43 Hz, furan), 9.05 (s, 1H, NH, D_2_O exchangeable), 9.52 (s, 1H, NH, D_2_O exchangeable); ^13^C NMR (DMSO-*d*_6_) *δ* 20.2 (1C, CH_3_), 31.4, 54.1 (2C, 2CH, pyran ring), 60.8 (1C, OCH_3_), 89.2 (1C, CH-pyran ring), 92.1 (1C, CH, benzene), 102.8, 104.5, 106.2, 127.7, 128.4, 129.6, 132.9, 146.3, 152.5, 153.9, 155.7, 156.8 (14C, Ar-C), 159.1 (1C, C=O); MS (70 eV, %) *m*/*z* 377 (M^+^, 72%); Anal. Calc. (Found) for C_21_H_19_N_3_O_4_ (377.40): C, 66.83 (66.75); H, 5.07 (5.15); N, 11.13 (11.05). 

### 3.42. Synthesis of 4-imino-6,10-dimethoxy-5-methyl-3-phenyl-1,3,4,4a,5,11a-hexahydro-2H-furo [3′,2′:6,7]chromeno[2,3-d]pyrimidin-2-one (***16b***)

The compound was obtained from the reaction of (**4b**) (2.88 g, 0.01 mol) and phenylisocyanate as pale yellow crystals, crystallized from benzene (53%), M.p. > 350 °C. IR (*ν*, cm^−1^) KBr: broad 3282–3233 (2NH), 3073 (CH-aryl), 2960 (CH-aliph), 1682 (C=O), 1633 (C=N), 1581 (C=C), ^1^H NMR (DMSO-*d*_6_, ppm) *δ* 1.32 (d, 3H, *J* = 6.73 Hz, CH_3_), 3.14 (m, 1H, CH_,_ pyran ring), 3.35 (t, 1H, *J* = 6.70 Hz, CH_,_ pyran ring), 3.95 (s, 6H, 2OCH_3_), 5.30 (d, 1H, *J* = 6.72 Hz, CH_,_ pyran ring), 6.82 (d, 1H, *J* = 2.40 Hz, furan), 7.45–7.65 (s, 5H, phenyl ring), 7.72 (d, 1H, *J* = 2.44 Hz, furan), 9.17 (s, 1H, NH, D_2_O exchangeable), 9.50 (s, 1H, NH, D_2_O exchangeable); ^13^C NMR (DMSO-*d*_6_) *δ* 20.5 (1C, CH_3_), 31.7, 54.5 (2C, 2CH, pyran ring), 61.1 (2C, 2OCH_3_), 90.3 (1C, CH-pyran ring), 105.5, 106.2, 110.4, 126.8, 127.9, 128.5, 129.2, 133.1, 144.8, 145.7, 146.3, 146.8, 156.7 (15C, Ar-C), 160.5 (1C, C=O); MS (70 eV, %) *m*/*z* 407 (M^+^, 69%); Anal. Calc. (Found) for C_22_H_21_N_3_O_5_ (407.43): C, 64.86 (64.80); H, 5.20 (5.12); N, 10.31 (10.38).

### 3.43. Synthesis of Ethyl -N-(6-cyano-(4-methoxy or 4, 9-dimethoxy)-5-methyl-6,7-dihydro-5H- furo[3,2-g]chromen-7-yl)formimidate (***17a,b***)

General procedure: A mix of compound (**4a**) (2.58 g, 0.01 mol) or (**4b**) (2.88 g, 0.01 mol) and triethyl-orthoformate (1.50 mL, 0.01 mol) and acetic anhydride (40 mL) was refluxed for 5–8 h under control (TLC). The solvent was removed under reduced pressure and the separated solid was recrystallized from the proper solvent to give (**17a**) or (**17b**)**.**

### 3.44. Synthesis of Ethyl-N-(6-cyano-4-methoxy-5-methyl-6,7-dihydro-5H-furo[3,2-g]chromen -7-yl) formimidate (***17a***)

The compound was obtained from the reaction of (**4a**) (2.58 g, 0.01mol) and triethyl-orthoformate as brownish crystals, crystallized from methanol (73%), M.p.: 270–272 °C. IR (*ν*, cm^−1^) KBr: 3049 (CH-aryl), 2955 (CH-aliph), 2220 (CN), 1635(C=N), 1580 (C=C). ^1^H NMR (DMSO-*d*_6_, ppm) *δ* 1.25 (t, 3H, *J* = 6.72 Hz, CH_3_), 1.35 (d, 3H, *J* = 6.75 Hz, CH_3_), 3.27 (m, 1H, *J* = 6.80 Hz, CH_,_ pyran ring), 3.35 (t, 1H, *J* = 6.85 Hz, CH_,_ pyran ring), 3.39 (d, 1H, *J* = 6.68 Hz, CH_,_ pyran ring), 3.66 (q, 2H, *J* = 6.65 Hz, CH_2_), 3.80 (s, 3H, OCH_3_), 6.82 (d, 1H, *J* = 2.33 Hz, furan), 7.17 (s, 1H, benzene), 7.50 (d, 1H, *J* = 2.46 Hz, furan), 8.02 (s, 1H,CH, methine proton); ^13^C NMR (DMSO-*d*_6_) *δ* 18.5, 20.7 (2C, 2CH_3_), 21.5, 45.2 (2C, 2CH, pyran ring), 60.8 (1C, OCH_3_), 64.2 (1C, CH_2_), 90.6 (1C, benzene ring), 92.3 (1C, pyran ring), 119.5 (1C, CN), 103.6, 105.1, 106.3, 146.5, 152.1, 154.7, 155.6 (7C, Ar-C), 159.1 (1C,C=N); MS (70 eV, %) *m*/*z* 314 (M^+^, 100%); Anal. Calc. (Found) for C_17_H_18_N_2_O_4_ (314.34): C, 64.96 (64.88); H, 5.77 (5.70); N, 8.91 (8.98).

### 3.45. Synthesis of Ethyl-N-(6-cyano-4,9-dimethoxy-5-methyl-6,7-dihydro-5H-furo[3,2-g] chromen-7-yl)formimidate (***17b***)

The compound was obtained from the reaction of (**4b**) (2.88 g, 0.01mol) and triethyl-orthoformate as yellowish crystals, crystallized from acetone (67%), M.p.: 280–282 °C. IR (*ν*, cm^−1^) KBr: 3045 (CH-aryl), 2951 (CH-aliph), 2218 (CN), 1633 (C=N), 1583 (C=C). ^1^H NMR (DMSO-*d*_6_, ppm) *δ* 1.20 (t, 3H, *J* = 6.68 Hz, CH_3_), 1.31 (d, 3H, *J* = 6.69 Hz, CH_3_), 3.23 (m, 1H, *J* = 6.73 Hz, CH_,_ pyran ring), 3.29 (t, 1H, *J* = 6.76 Hz, CH_,_ pyran ring), 3.37 (d, 1H, *J* = 6.74 Hz, CH_,_ pyran ring), 3.61 (q, 2H, *J* = 6.67 Hz, CH_2_), 3.91 (s, 6H, 2OCH_3_), 6.85 (d, 1H, *J* = 2.35 Hz, furan), 7.55 (d, 1H, *J* = 2.44 Hz, furan), 8.06 (s, 1H,CH, methine proton); ^13^C NMR (DMSO-*d*_6_) *δ* 19.2, 20.9 (2C, 2CH_3_), 21.8, 45.7 (2C, 2CH, pyran ring), 62.5 (2C, 2OCH_3_), 64.6 (1C, CH_2_), 91.9 (1C, pyran ring), 119.7 (1C, CN), 106.1, 106.7, 110.5, 127.1, 145.1, 145.8, 146.3, 146.9 (8C, Ar-C), 159.7 (1C,C=N); MS (70 eV, %) *m*/*z* 344 (M^+^, 97%); Anal. Calc. (Found) for C_18_H_20_N_2_O_5_ (344.37): C, 62.78 (62.85); H, 5.85 (5.77); N, 8.13 (8.21).

### 3.46. Synthesis of (6-methoxy or 6,10-dimethoxy)-3,5-dimethyl-3,4a,5,11a-tetrahydro-4H-furo [3′,2′:6,7]chromeno[2,3-d]pyrimidin-4-imine (***18a,b***)

General procedure: A mix of compound (**17a**) (3.14 g, 0.01 mol) or (**17b**) (3.44 g, 0.01 mol) and methylamine (0.04 mL, 0.01 mol) in absolute ethanol (40 mL) was heated and stirred at room temperature for 2–4 h with TLC. The resulting product was collected via filtration and recrystallized from the appropriate solvent to give (**18a**) or (**18b**).

### 3.47. Synthesis of 6-methoxy-3,5-dimethyl-3,4a,5,11a-tetrahydro-4H-furo[3′,2′:6,7]chromeno[2, 3-d]pyrimidin -4-imine (***18a***)

The compound was obtained from the reaction of (**17a**) (3.14 g, 0.01 mol) and methylamine as white crystals, crystallized from dioxane (82%), M.p. > 350 °C. IR (*ν*, cm^−1^) KBr: broad 3250 (NH), 3045 (CH-aryl), 2960 (CH-aliph), 1636 (C=N), 1589 (C=C), ^1^H NMR (DMSO-*d*_6_, ppm) *δ* 1.25 (d, 3H, *J* = 6.85 Hz, CH_3_), 3.23 (m, 1H, CH_,_ pyran ring), 3.30 (t, 1H, *J* = 6.88 Hz, CH_,_ pyran ring), 3.38 (s, 3H, CH_3_), 3.80 (s, 3H, OCH_3_), 5.50 (d, 1H, *J* = 6.87 Hz, CH_,_ pyran ring), 6.82 (d, 1H, *J* = 2.44 Hz, furan), 7.30 (s, 1H, benzene), 7.70 (s, 1H, pyrimidine ring), 7.80 (d, 1H, *J* = 2.43 Hz, furan), 9.65 (s, 1H, NH, D_2_O exchangeable); ^13^C NMR (DMSO-*d*_6_) *δ* 20.4, 27.8 (2C, 2CH_3_), 29.5, 52.4 (2C, 2CH, pyran ring), 60.9 (1C, OCH_3_), 90.1 (1C, CH-pyran ring), 91.7 (1C, CH, benzene), 103.6, 105.5, 106.7, 146.4, 152.1, 152.7, 154.3, 155.8, 157.6 (9C, Ar-C); MS (70 eV, %) *m*/*z* 299 (M^+^, 98%); Anal. Calc. (Found) for C_16_H_17_N_3_O_3_ (299.33): C, 64.20 (64.29); H, 5.72 (5.65); N, 14.04 (14.10).

### 3.48. Synthesis of 6,10-dimethoxy-3,5-dimethyl-3,4a,5,11a-tetrahydro-4H-furo[3′,2′:6,7] chromeno [2,3-d]pyrimidin-4-imine (***18b***)

The compound was obtained from the reaction of (**17b**) (3.44 g, 0.01 mol) and methylamine as brownish crystals, crystallized from DMF (78%), M.p. > 350 °C. IR (*ν*, cm^−1^) KBr: broad 3245 (NH), 3048 (CH-aryl), 2967 (CH-aliph), 1632 (C=N), 1581 (C=C), ^1^H NMR (DMSO-*d*_6_, ppm) *δ* 1.28 (d, 3H, *J* = 6.87 Hz, CH_3_), 3.26 (m, 1H, CH_,_ pyran ring), 3.35 (t, 1H, *J* = 6.86 Hz, CH_,_ pyran ring), 3.40 (s, 3H, CH_3_), 3.93 (s, 6H, 2OCH_3_), 5.58 (d, 1H, *J* = 6.82 Hz, CH_,_ pyran ring), 6.85 (d, 1H, *J* = 2.41 Hz, furan), 7.66 (s, 1H, pyrimidine ring), 7.85 (d, 1H, *J* = 2.40 Hz, furan), 9.62 (s, 1H, NH, D_2_O exchangeable); ^13^C NMR (DMSO-*d*_6_) *δ* 20.6, 28.1 (2C, 2CH_3_), 30.2, 52.7 (2C, 2CH, pyran ring), 61.8 (2C, 2OCH_3_), 90.5 (1C, CH-pyran ring), 105.4, 106.1, 110.6, 127.2, 145.1, 145.7, 146.2, 147.1, 152.5, 156.9 (10 C, Ar-C); MS (70 eV, %) *m*/*z* 329 (M^+^, 95%); Anal. Calc. (Found) for C_17_H_19_N_3_O_4_ (329.36): C, 62.00 (62.10); H, 5.81 (5.75); N, 12.76 (12.70).

### 3.49. Synthesis of 4-imino-(6-methoxy or 6,10-dimethoxy)-5-methyl-4a, 11a-dihydro-4H-furo [3′, 2′:6,7]chromeno [2,3-d]pyrimidin-3(5H)-amine (***19a,b***)

General procedure: A mixture of compound (**17a**) (3.14 g, 0.01 mol) or (**17b**) (3.44 g, 0.01 mol) and hydrazine hydrate (1 mL, excess) was created in absolute ethanol (45 mL). The reaction solution was refluxed for 3–5 h with TLC, the reaction mixture was concentrated, and the precipitate product that separated out was filtered off and recrystallized from the suitable solvent to give (**19a**) or (**19b**).

### 3.50. Synthesis of 4-imino-6-methoxy-5-methyl-4a, 11a-dihydro-4H-furo [3′,2′: 6,7]chromeno [2, 3-d]pyrimidin-3(5H)-amine (***19a***)

The compound was obtained from the reaction of (**17a**) (3.14 g, 0.01 mol) and hydrazine hydrate as yellowish crystals, crystallized from methanol (76%), M.p. > 350 °C. IR (*ν*, cm^−1^) KBr: broad 3425 (NH_2_), 3260 (NH), 3052 (CH-aryl), 2964 (CH-aliph), 1637 (C=N), 1590 (C=C), ^1^H NMR (DMSO-*d*_6_, ppm) *δ* 1.27 (d, 3H, *J* = 6.78 Hz, CH_3_), 3.31 (m, 1H, CH_,_ pyran ring), 3.39 (t, 1H, *J* = 6.90 Hz, CH_,_ pyran ring), 3.84 (s, 3H, OCH_3_), 5.60 (d, 1H, *J* = 6.91 Hz, CH_,_ pyran ring), 6.40 (s, 2H, NH_2,_ D_2_O exchangeable), 6.73 (d, 1H, *J* = 2.41 Hz, furan), 7.26 (s, 1H, benzene), 7.65 (s, 1H, pyrimidine ring), 7.77 (d, 1H, *J* = 2.40 Hz, furan), 9.70 (s, 1H, NH, D_2_O exchangeable); ^13^C NMR (DMSO-*d*_6_) *δ* 19.4 (1C, CH_3_), 26.9, 51.2 (2C, 2CH, pyran ring), 60.2 (1C, OCH_3_), 85.5 (1C, CH-pyran ring), 91.1 (1C, CH, benzene), 103.8, 105.4, 106.5, 146.2, 147.3, 152.6, 154.1, 155.9, 156.8 (9C, Ar-C); MS (70 eV, %) *m*/*z* 300 (M^+^, 100%); Anal. Calc. (Found) for C_15_H_16_N_4_O_3_ (300.32): C, 59.99 (59.90); H, 5.37 (5.45); N, 18.66 (18.58).

### 3.51. Synthesis of 4-imino-6,10-dimethoxy-5-methyl-4a,11a-dihydro-4H-furo [3′,2′:6,7] chromeno [2,3-d]pyrimidin-3(5H)-amine (***19b***)

The compound was obtained from the reaction of (**17b**) (3.44 g, 0.01 mol) and hydrazine hydrate as yellow crystals, crystallized from ethyl-acetate (71%), M.p. > 350 °C. IR (*ν*, cm^−1^) KBr: broad 3422 (NH_2_), 3258 (NH), 3051 (CH-aryl), 2962 (CH-aliph), 1633 (C=N), 1581 (C=C), ^1^H NMR (DMSO-*d*_6_, ppm) *δ* 1.25 (d, 3H, *J* = 6.76 Hz, CH_3_), 3.29 (m, 1H, CH_,_ pyran ring), 3.41 (t, 1H, *J* = 6.87 Hz, CH_,_ pyran ring), 3.94 (s, 6H, 2OCH_3_), 5.68 (d, 1H, *J* = 6.85 Hz, CH_,_ pyran ring), 6.35 (s, 2H, NH_2,_ D_2_O exchangeable), 6.70 (d, 1H, *J* = 2.42 Hz, furan), 7.61 (s, 1H, pyrimidine ring), 7.85 (d, 1H, *J* = 2.39 Hz, furan), 9.75 (s, 1H, NH, D_2_O exchangeable); ^13^C NMR (DMSO-*d*_6_) *δ* 20.2 (1C, CH_3_), 28.3, 52.4 (2C, 2CH, pyran ring), 61.9 (2C, 2OCH_3_), 86.1 (1C, CH-pyran ring), 106.1, 106.8, 110.3, 126.8, 145.5, 146.1, 146.7, 147.4, 147.7, 157.2 (10C, Ar-C); MS (70 eV, %) *m*/*z* 330 (M^+^, 90%); Anal. Calc. (Found) for C_16_H_18_N_4_O_4_ (330.34): C, 58.17 (58.25); H, 5.49 (5.41); N, 16.96 (16.88).

### 3.52. Synthesis of (12-methoxy or 8,12-dimethoxy)-13-methyl-6a,13a-dihydro-13H-furo [3′,2′: 6, 7]chromeno [3,2-e][1,2,4]triazolo [1,5-c]pyrimidine (***20a,b***)

General procedure: A mixture of compound (**19a**) (3.00 g, 0.01 mol) or (**19b**) (3.30 g, 0.01 mol), excess of triethyl-orthoformate (6 mL), and acetic anhydride (30 mL) was refluxed for 4–6 h under control (TLC). The solvent was removed under reduced pressure; the separated solid was filtered off and recrystallized from the proper solvent to give triazolopyrimidines (**20a**) and (**20b**)**.**

### 3.53. Synthesis of 12-methoxy-13-methyl-6a,13a-dihydro-13H-furo [3′,2′:6,7]chromeno [3,2-e][1,2,4]triazolo [1,5-c]pyrimidine (***20a***)

The compound was obtained from the reaction of (**19a**) (3.00 g, 0.01 mol) and triethylorthoformate as brownish crystals, crystallized from dioxane (68%), M.p. > 350 °C. IR (*ν*, cm^−1^) KBr: broad 3045 (CH-aryl), 2937 (CH-aliph), 1633 (C=N), 1582 (C=C), ^1^H NMR (DMSO-*d*_6_, ppm) *δ* 1.23 (d, 3H, *J* = 6.80 Hz, CH_3_), 3.22 (m, 1H, CH_,_ pyran ring), 3.31 (t, 1H, *J* = 6.87 Hz, CH_,_ pyran ring), 3.82 (s, 3H, OCH_3_), 5.40 (d, 1H, *J* = 6.83 Hz, CH_,_ pyran ring), 6.10 (s, 1H,CH, triazole ring), 6.71 (d, 1H, *J* = 2.35 Hz, furan), 7.15 (s, 1H, benzene), 7.72 (s, 1H, pyrimidine ring), 7.83 (d, 1H, *J* = 2.33 Hz, furan); ^13^C NMR (DMSO-*d*_6_) *δ* 21.8 (1C, CH_3_), 27.3, 52.7 (2C, 2CH, pyran ring), 60.6 (1C, OCH_3_), 90.8 (1C, CH, benzene), 95.2 (1C, CH-pyran ring), 102.9, 105.1, 106.3, 139.7, 146.5, 151.4, 152.2, 152.6, 154.1, 156.2 (10C, Ar-C); MS (70 eV, %) *m*/*z* 310 (M^+^, 85%); Anal. Calc. (Found) for C_16_H_14_N_4_O_3_ (310.31): C, 61.93 (61.84); H, 4.55 (4.63); N, 18.06 (18.14).

### 3.54. Synthesis of 8,12-dimethoxy-13-methyl-6a,13a-dihydro-13H-furo [3′,2′:6,7]chromeno [3,2-e] [1,2,4]triazolo [1, 5-c]pyrimidine (***20b***)

The compound was obtained from the reaction of (**19b**) (3.30 g, 0.01 mol) and triethylorthoformate as pale brown crystals, crystallized from DMF (64%), M.p. > 350 °C. IR (*ν*, cm^−1^) KBr: broad 3050 (CH-aryl), 2940 (CH-aliph), 1630 (C=N), 1580 (C=C), ^1^H NMR (DMSO-*d*_6_, ppm) *δ* 1.28 (d, 3H, *J* = 6.84 Hz, CH_3_), 3.26 (m, 1H, CH_,_ pyran ring), 3.37 (t, 1H, *J* = 6.85 Hz, CH_,_ pyran ring), 3.90 (s, 6H, 2OCH_3_), 5.45 (d, 1H, *J* = 6.81 Hz, CH_,_ pyran ring), 6.05 (s, 1H,CH, triazole ring), 6.88 (d, 1H, *J* = 2.31 Hz, furan), 7.76 (s, 1H, pyrimidine ring), 7.88 (d, 1H, *J* = 2.37 Hz, furan); ^13^C NMR (DMSO-*d*_6_) *δ* 21.2 (1C, CH_3_), 27.8, 53.1 (2C, 2CH, pyran ring), 62.3 (2C, 2OCH_3_), 99.1 (1C, CH-pyran ring), 105.5, 106.4, 110.6, 126.8, 139.9, 145.2, 146.1, 146.6, 147.3, 151.5, 152.7 (11C, Ar-C); MS (70 eV, %) *m*/*z* 340 (M^+^, 80%); Anal. Calc. (Found) for C_17_H_16_N_4_O_4_ (340.34): C, 60.00 (60.10); H, 4.74 (4.66); N, 16.46 (16.55).

### 3.55. Synthesis of (4-methoxy or 4,13-dimethoxy)-5-methyl-5a,7,8,9,10,11a-hexahydro-5H-furo [3′,2′:6,7]chromeno [2,3-b]quinolin-6-amine (***21a,b***)

General procedure for the preparation [37]: aluminum chloride (1.33 g, 0.01 mol) was suspended in dry 1, 2-dichloroethane (20 mL) at room temperature under an argon atmosphere. After stirring the suspension for a few minutes, the corresponding compound, (**4a**) (2.58 g, 0.01 mol) or (**4b**) (2.88 g, 0.01 mol), and cyclohexanone (1.03 mL, 0.01 mol) were added to the mixture and the reaction mixture was heated under reflux for 23–27 h. The reaction was monitored via TLC. After accomplishment of the reaction, an aqueous solution of sodium hydroxide (10%) was added dropwise to the mixture until the aqueous solution became basic. After stirring for 60 min, the final precipitate was filtered, washed with water, and recrystallized from the suitable solvent to give (**21a**) or (**21b**). 

### 3.56. Synthesis of 4-methoxy-5-methyl-5a,7,8,9,10,11a-hexahydro-5H-furo [3′,2′:6,7]chromeno [2, 3-b]quinolin-6-amine (***21a***)

The compound was obtained from the reaction of (**4a**) (2.58 g, 0.01 mol) and cyclohexanone as pale yellow crystals, crystallized from DMF (75%), M.p. > 350 °C. IR (*ν*, cm^−1^) KBr: broad 3415 (NH_2_), 3057 (CH-aryl), 2963 (CH-aliph), 1638 (C=N), 1589 (C=C). ^1^H NMR (DMSO-*d*_6_, ppm) *δ* 1.32 (d, 3H, *J* = 6.83 Hz, CH_3_), 1.45–2.25 (m, 8H, cyclohexane ring), 3.30 (m, 1H, *J* = 6.85 Hz, CH, pyran ring), 3.40 (t, 1H, *J* = 6.88 Hz, CH_,_ pyran ring), 3.87 (s, 3H, OCH_3_), 5.38 (d, 1H, *J* = 6.68 Hz, CH_,_ pyran ring), 6.65 (s, 2H, NH_2,_ D_2_O exchangeable), 6.88 (d, 1H, *J* = 2.37 Hz, furan), 7.29 (s, 1H, benzene), 7.70 (d, 1H, *J* = 2.39 Hz, furan); ^13^C NMR (DMSO-*d*_6_) *δ* 19.1 (1C, CH_3_), 22.3, 25.5, 26.8, 30.9 (4C, 4CH_2,_ cyclohexane ring), 31.6, 55.5 (2C, 2CH, pyran ring), 60.7 (1C, OCH_3_), 90.8 (1C, benzene ring), 92.1, 98.4 (2C, pyridine ring), 103.4, 105.2, 106.1, 146.7, 152.5, 153.3, 154.1, 156.5, 165.2 (9C, Ar-C); MS (70 eV, %) *m*/*z* 338 (M^+^, 93%); Anal. Calc. (Found) for C_20_H_22_N_2_O_3_ (338.41): C, 70.99 (70.90); H, 6.55 (6.63); N, 8.28 (8.21).

### 3.57. Synthesis of 4,13-dimethoxy-5-methyl-5a,7,8,9,10,11a-hexahydro-5H-furo [3′,2′:6,7] chromeno [2,3-b]quinolin-6-amine (***21b***)

The compound was obtained from the reaction of (**4b**) (2.88 g, 0.01 mol) and cyclohexanone as yellowish crystals, crystallized from dioxane (73%), M.p. > 350 °C. IR (*ν*, cm^−1^) KBr: broad 3412 (NH_2_), 3059 (CH-aryl), 2966 (CH-aliph), 1631 (C=N), 1582 (C=C). ^1^H NMR (DMSO-*d*_6_, ppm) *δ* 1.26 (d, 3H, *J* = 6.79 Hz, CH_3_), 1.47–2.27 (m, 8H, cyclohexane ring), 3.35 (m, 1H, *J* = 6.75 Hz, CH, pyran ring), 3.44 (t, 1H, *J* = 6.77 Hz, CH_,_ pyran ring), 3.91 (s, 6H, 2OCH_3_), 5.41 (d, 1H, *J* = 6.74 Hz, CH_,_ pyran ring), 6.71 (s, 2H, NH_2,_ D_2_O exchangeable), 6.90 (d, 1H, *J* = 2.35 Hz, furan), 7.75 (d, 1H, *J* = 2.31 Hz, furan); ^13^C NMR (DMSO-*d*_6_) *δ* 19.4 (1C, CH_3_), 22.5, 25.8, 27.1, 31.3 (4C, 4CH_2,_ cyclohexane ring), 32.2, 55.8 (2C, 2CH, pyran ring), 61.9 (2C, 2OCH_3_), 92.6, 98.7 (2C, pyridine ring), 105.3, 105.7, 110.5, 127.5, 145.2, 145.5, 146.4, 146.8, 153.5, 165.7 (10C, Ar-C); MS (70 eV, %) *m*/*z* 368 (M^+^, 90%); Anal. Calc. (Found) for C_21_H_24_N_2_O_4_ (368.43): C, 68.46 (68.52); H, 6.57 (6.50); N, 7.60 (7.68).

### 3.58. Biological Screening (Materials and Methods, In Vitro)

The antimicrobial activity of the newly prepared compounds was tested in vitro against Gram-negative bacteria *Klebsiella pneumoniae* (ATCC^®^ 10031™) and *Escherichia coli* (ATCC^®^ 25922™); Gram-positive bacteria *Streptococcus pyogenes* (ATCC^®^ 19615™) and *Staphylococcus aureus* (ATCC^®^ 6538™); and the fungi *Candida albicans* (ATCC^®^ 10231™), *Curvularia lunata, Alternaria alternate*, and *Aspergillus niger* (ATCC^®^ 16888™). The newly synthesized compounds were dissolved in dimethyl sulfoxide (DMSO) and tested for their antimicrobial activity by the agar disk diffusion technique. Cefotaxime sodium and nystatin [9,10,33,34,35,38,39,40,41,42,43,44,45,46,47,48,49,50,51,52,53,54] were used as standard drugs for the antibacterial and antifungal assays, respectively. A solution of 100 μg mL^−1^ of the tested compound and microplate wells 1 cm in diameter were used. Zones of inhibition were measured with calipers or automated scanners and paralleled with those of the standards. Cefotaxime sodium (0.15 μmol mL^−1^) and nystatin (0.037 μmol mL^−1^) were used as the standard drugs for antibacterial and antifungal activity, respectively. Compound-impregnated disks were placed on an agar plate containing a standard suspension of microorganisms. The plate was incubated for 24 h at 37 °C. For the assessment of the minimum inhibitory concentration (MIC) by serial plate dilution [9,10,33,34,35,38,39,40,41,42,43,44,45,46,47,48,49,50,51,52,53,54], 5 mg of each tested compound was dissolved in 1 mL of DMSO separately to prepare stock solutions. Serial dilutions were prepared from each stock solution. The plates were incubated at 37 °C for 24 h. MIC is defined as the lowest concentration (μmol mL^−1^) of the tested compound that results in no visible growth on the plates. DMSO was used as the solvent control to ensure that the solvent had no effect on bacterial growth. The results are shown in Table 1 and Table 2.

#### 3.58.1. Ethical Approval and Consent to Participate

No humans or animals were used in this study; nevertheless, all the procedures were carried out under the approval of the Medical Research Ethics Committee of the National Research Centre, Department of Chemistry of Natural and Microbial Products, Giza 12622, Egypt.

#### 3.58.2. Human and Animal Rights

No human or animal subjects were used in the study. The research was conducted according to ethical standards in vitro.

#### 3.58.3. Chemicals and Drugs

Types of Gram-positive bacteria Staphylococcus aureus and Streptococcus pyogenes, Gram-negative bacteria Escherichia coli and Klebsiella pneumoniae, and fungi Aspergillus niger, Alternaria alternate, Curvularia lunata, and Candida albicans were from the National Research Centre, Department of Chemistry of Natural and Microbial Products, Giza, Egypt, and cefotaxime sodium, nystatin, and DMSO were purchased from Sigma-Aldrich.

## 4. Conclusions

In the present research, the furochromone ring system is confirmed to be one of the most significant heterocyclic compounds in nature. It is found in neurotransmitters such as serotonin and complex alkaloids such as the Khellol glucoside, Bergapten, Ricchiocarpen, and chromenes. Likewise, a number of important synthetic drugs contain a chromene ring. Therefore, we prepared new heterocyclic compounds of furo [3,2-*g*] chromene -6-carbonitrile such as furochromeno [2,3-*d*]pyrimidines ((**5**), (**6**), (**8**), (**11**), (**12**), (**14–16**), (**18**), (**19**)), *N*-(6-cyano-5-methyl-furo [3,2-*g*]chromene) acetamide (**7**), *N*-(6-cyano- 5-methyl-furo[3,2-*g*]chromene)-phenylacetamide (**13**), *N*-(6-cyano-furo[3,2-*g*]chromene) formimidate (**17**), furochromeno [2,3-e][1,2,4]triazepin-amine (9), furo[3,2-g]chromene- 6-carboxamide (10), furochromeno [3,2-e][1,2,4]triazolopyrimidines (**20**), and furo- chromeno[2,3-*b*]quinolin-6-amine (**21**) derivatives; these compounds were tested as antimicrobials in vitro and studied in terms of molecular docking. From observations of the biological assay data and the molecular docking results, we concluded that the antibacterial activities of these compounds are clearly derived from the interaction between the compounds and the amino acid of the protein molecule (enzyme FabH). Hence, these compounds—furochromenotriazolopyrimidine (**20a**, **b**), furochromeno- quinoline (**21a**, **b**), furochromenotriazepine (**9a**, **b**) and furochromenopyrimidine (**19a**, **b**)—have potential for inhibiting microbial growth. Furthermore, we confirmed by this study that compounds (**20b**) and (**21b**) are promising antimicrobial agents and could be used for treating a selected range of microbial infections.

## Figures and Tables

**Table 1:** A minimum inhibitory concentration of the compounds against bacteria.

MIC (µmol mL^−1^)				
Compounds	Microorganisms			
	Gram-Negative Bacteria		Gram-Positive Bacteria	
	*Klebsiella pneumoniae*	*E. coli*	*Streptococcus pyogenes*	*Staphylococcus aureus*
**(3a)**	36	37	38	39
**(3b)**	35	36	37	38
**(4a)**	34	35	36	37
**(4b)**	33	34	35	36
**(5a)**	16	17	18	19
**(5b)**	15	16	17	18
**(6a)**	22	23	24	25
**(6b)**	21	22	23	24
**(7a)**	32	33	34	35
**(7b)**	31	32	33	34
**(8a)**	24	25	26	27
**(8b)**	23	24	25	26
**(9a)**	5	6	6	7
**(9b)**	4	4	5	6
**(10a)**	26	27	28	29
**(10b)**	25	26	27	28
**(11a)**	20	21	22	23
**(11b)**	19	20	21	22
**(12a)**	14	15	16	17
**(12b)**	13	13	14	15
**(13a)**	28	29	30	31
**(13b)**	27	28	29	30
**(14a)**	18	19	20	21
**(14b)**	17	18	19	20
**(15a)**	8	9	9	10
**(15b)**	7	8	8	9
**(16a)**	10	10	11	12
**(16b)**	9	10	11	11
**(17a)**	30	31	32	33
**(17b)**	29	30	31	32
**(18a)**	12	13	14	14
**(18b)**	11	12	13	13
**(19a)**	6	7	8	9
**(19b)**	5	6	7	8
**(20a)**	2	3	4	5
**(20b)**	1	2	3	4
**(21a)**	3	4	5	6
**(21b)**	2	3	4	5
**Cefotaxime sodium**	1	2	3	4
**Negative** **control**	NI	NI	NI	NI

DMSO was used as the negative control and as the solvent for test compounds and the reference drug.

**Table 2:** A minimum inhibitory concentration of the compounds against fungi.

MIC (µmol mL^−1^)				
Compounds	Microorganisms			
	*Candida albicans*	*Curvularia lunata*	*Alternaria alternata*	*Aspergillus niger*
**(3a)**	34	35	36	37
**(3b)**	33	34	35	36
**(4a)**	32	33	34	35
**(4b)**	31	32	33	34
**(5a)**	14	15	16	17
**(5b)**	13	14	15	16
**(6a)**	20	21	22	23
**(6b)**	19	20	21	22
**(7a)**	30	31	32	33
**(7b)**	29	30	31	32
**(8a)**	22	23	24	25
**(8b)**	21	22	23	24
**(9a)**	3	4	5	6
**(9b)**	2	3	4	5
**(10a)**	24	25	26	27
**(10b)**	23	24	25	26
**(11a)**	18	19	20	21
**(11b)**	17	18	19	20
**(12a)**	12	13	14	15
**(12b)**	11	12	13	14
**(13a)**	26	27	28	29
**(13b)**	25	26	27	28
**(14a)**	16	17	18	19
**(14b)**	15	16	17	18
**(15a)**	6	6	7	8
**(15b)**	5	6	6	7
**(16a)**	8	9	9	10
**(16b)**	7	8	8	9
**(17a)**	28	29	30	31
**(17b)**	27	28	29	30
**(18a)**	10	11	12	13
**(18b)**	9	10	11	12
**(19a)**	4	5	6	7
**(19b)**	3	4	5	6
**(20a)**	2	3	4	5
**(20b)**	1	2	2	3
**(21a)**	2	2	3	4
**(21b)**	1	1	2	3
**Nystatin**	1	1	2	3
**Negative** **control**	NI	NI	NI	NI

DMSO was used as the negative control and as the solvent for test compounds and the reference drug.

**Table 3:** Glide score, glide energy, binding energies (MM/GBSA), and interaction of the synthesized molecules with amino acid residues of 1HNJ protein.

Compounds	Structure	Glide Score	MM-GBSA	Protein–Ligand Interactions	Bond Distance °A
**(9a)**	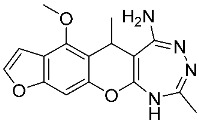	−4.322	−40.16	ARG151–Pyran	4.87
				ARG36–Pyran	5.50
				H_2_O–O (HB)	1.57
				H_2_O–NH_2_	2.06
				TRP32–Furan	3.64
				TRP32–Ph	3.57
**(9b)**	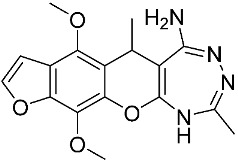	−3.17	−47.31	ASN210–O	1.71
**(19a)**	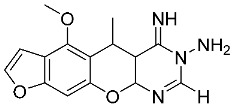	−5.181	−47.29	ALA246–NH_2_	2.15
				H_2_O–NH	1.76
				H_2_O–O	1.74
				ARG36–Ph	4.09
**(19b)**	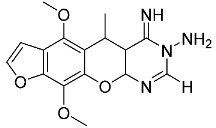	−3.055	−38.8	H_2_O–NH	2.17
				H_2_O–O_pyran	1.88
				H_2_O–OCH_3_	1.80
**(20a)**	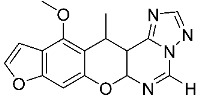	−6.799	−41.41	ASN274–O furan	2.3
**(20b)**	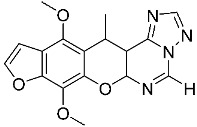	−3.248	−42.40	H_2_O–N pyrimidine	1.94
				H_2_O–OCH_3_	1.75
				TRP32–Ph	5.19
**(21a)**	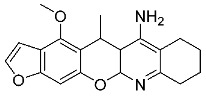	−3.992	−40.07	ASN–O Furan	2.01
				H_2_O–O pyran	2.06
				H_2_O–N pyrodine	1.9
				H_2_O–OCH_3_	1.85
**(21b)**	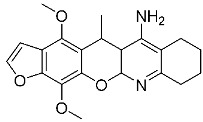	−5.833	−49.84	ARG36–NH_2_	2.52
				ARG36–OCH_3_	2.48
				H_2_O–O pyran	1.87
				ASN210–OCH_3_	2.49

## Data Availability

All data are contained within the article.
